# Minoxidil restores thymic growth in 22q11.2 deletion syndrome by limiting Sox9^+^ chondrocyte expansion

**DOI:** 10.70962/jhi.20250143

**Published:** 2025-08-12

**Authors:** Pratibha Bhalla, Neha Ahuja, Ashwani Kumar, Chao Xing, Angela Moses, Ashutosh Shukla, Katelyn Boetel, Bret M. Evers, John M. Shelton, Maria Teresa de la Morena, Christian A. Wysocki, Ondine B. Cleaver, Nicolai S.C. van Oers

**Affiliations:** 1 Department of Immunology, The University of Texas Southwestern Medical Center, Dallas, TX, USA; 2 Molecular Biology, The University of Texas Southwestern Medical Center, Dallas, TX, USA; 3 Eugene McDermott Center for Human Growth and Development, The University of Texas Southwestern Medical Center, Dallas, TX, USA; 4 Department of Bioinformatics, The University of Texas Southwestern Medical Center, Dallas, TX, USA; 5 Population and Data Sciences, The University of Texas Southwestern Medical Center, Dallas, TX, USA; 6 Department of Internal Medicine, The University of Texas Southwestern Medical Center, Dallas, TX, USA; 7 Department of Pathology, The University of Texas Southwestern Medical Center, Dallas, TX, USA; 8 Division of Immunology, Department of Pediatrics, University of Washington, Seattle, WA, USA; 9 Seattle Children’s Hospital, Seattle, WA, USA; 10 Department of Pediatrics, The University of Texas Southwestern Medical Center, Dallas, TX, USA; 11 Department of Microbiology at The University of Texas Southwestern Medical Center, Dallas, TX, USA

## Abstract

Thymic hypoplasia, hypoparathyroidism, and cardiac defects are common congenital malformations caused by 22q11.2 deletion syndrome (22q11.2DS; aka DiGeorge syndrome). Thymus hypoplasia reduces peripheral T cell numbers, leading to more frequent infections. We report that embryonic hypoplastic thymuses from mouse models of 22q11.2DS (Tbx1^neo2/neo2^) have distinct mesenchymal cell subsets, including an expansion of Sox9^+^ chondrocytes. Chondrocytes produce collagens and extracellular matrix (ECM) proteins, which can affect thymus size and vascularization. Two compounds, minoxidil and PGE_2_, restored growth for Tbx1^neo2/neo2^ embryonic thymuses when administered to pregnant mice prior to formation of the thymic anlage. The dysregulation of the mesenchymal and endothelial transcriptomes was corrected with minoxidil in Tbx1^neo2/neo2^ thymuses. This was confirmed by the diminished expression of Sox9-driven type II, IX, and XI cartilaginous collagens and other ECM proteins. Furthermore, the location of parathyroids was corrected in Tbx1^neo2/neo2^ embryos. In summary, these findings reveal that targeting prenatal mesenchymal differentiation can correct multiple congenital anomalies in mouse models of 22q11.2DS.

## Teaser

22q11.2 deletion syndrome causes thymic hypoplasia. In an embryonic mouse model, Sox9-expressing chondrocytes expanded in the hypoplastic thymuses. A drug screen identified minoxidil as a therapeutic that restored thymus growth and limited the overproduction of collagens and ECM proteins when administered in pregnant mice.

## Graphical Abstract

**Figure:**
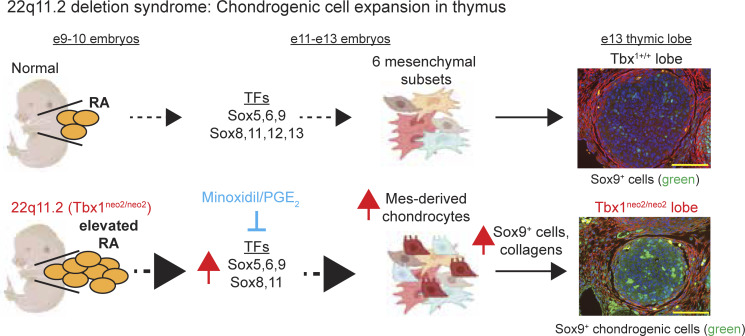

## Introduction

22q11.2 deletion syndrome (22q11.2DS) is the most common human chromosomal deletion syndrome known (1/2,150 live births) (1, 2). This deletion causes multiple congenital problems, including thymic hypoplasia, hypoparathyroidism, cardiac defects, and/or dysmorphic facial features. All are linked to developmental problems of the embryonic pharyngeal pouches and arch arteries (3, 4, 5, 6). Most 22q11.2DS individuals (85–90%) have a 3-Mb deletion on one allele of chromosome 22q11.2. This results in a haploinsufficiency of 46 protein-coding genes and ∼60 noncoding elements (3, 4, 5). The deletions are caused by misguided chromosomal recombination between four highly homologous low-copy repeats (LCR A–D) located within 22q11.2. LCRs are only found in higher-order primates, and humans having expanded their number and complexity to eight (LCR A–H), including multiple allelic variants of LCR A (7). The congenital defects in human 22q11.2DS have a noted variability in penetrance and are primarily linked to a haploinsufficiency of T-box transcription factor 1 (*TBX1*) encoded on 22q11.2 (3, 5, 6, 8, 9, 10, 11, 12, 13, 14, 15, 16, 17). Consistent with this transcription factor (TF) playing such a key role, both *TBX1* loss- and gain-of-function mutations can result in similar congenital abnormalities as 22q11.2DS (15, 16, 17, 18). Postnatal concerns for 22q11.2DS patients include immunodeficiency, cardiac issues, development delay, autism spectrum disorders, and schizophrenia (3, 5, 6, 19, 20). These postnatal problems are impacted by genes both within and outside the 22q11.2-deleted locus via direct and epigenetic processes (3, 20, 21, 22).

Thymus hypoplasia leading to low peripheral T cell numbers affects ∼60–70% of 22q11.2DS patients, a disorder first termed DiGeorge syndrome (5, 6, 23, 24, 25, 26, 27, 28). T cell lymphopenia results in patients having more frequent and severe infections, less effective vaccine responses, and higher incidences of autoimmunity (6, 8, 27, 29). Thymic aplasia is reported in <1% of 22q11.2DS patients (5). For these individuals, an allogeneic thymic stromal implant is a clinical treatment option (30, 31). In the Tbx1^neo2/neo2^ mouse model of 22q11.2DS, comparative single-cell RNA sequencing (scRNA Seq) revealed transcriptome differences among the embryonic mesenchymal subclusters and one endothelial population in the developing thymus (32). Increased collagen levels were evident in the smaller embryonic thymuses, confirmed in the postnatal thymuses from patients with 22q11.2DS (32). In a distinct 22q11.2DS mouse model (Tbx1^+/−^Crkl^+/−^), altered mesenchymal populations was also reported (33).

In the current study, we assessed the ability of different drugs to restore growth for the small thymuses. These were tested in fetal thymic organ cultures (FTOCs) and in utero in pregnant mouse models of 22q11.2DS. Two drugs administered in vivo, minoxidil and PGE_2_, re-established growth for the 22q11.2 embryonic thymuses and normalized the positioning of the parathyroids (Pth’s). Comparative scRNA Seq, immunofluorescence analyses, and whole-mount imaging revealed that hypoplastic thymuses from the Tbx1^neo2/neo2^ embryos had an overabundance of neural crest cell (NCC)–derived chondrocyte-type mesenchymal cells. These were particularly prominent in an expanded thymic capsule. The drug treatments reduced the trio of Sry-related HMG box (Sox)5, Sox6, and Sox9 TFs that drive chondrogenesis, resulting in normalization of collagen and extracellular matrix (ECM) transcripts in the thymuses. The findings establish a mechanistic basis for the congenital hypoplasia of the thymus in 22q11.2DS along with therapeutic strategies to correct these defects.

## Results

### Thymic mesenchymal cells from embryonic Tbx1^neo2/neo2^ 22q11.2 models exhibit elevated expression of chondrogenic-regulated collagens and Sox5, Sox6, and Sox9 TFs

**Figure S1.**
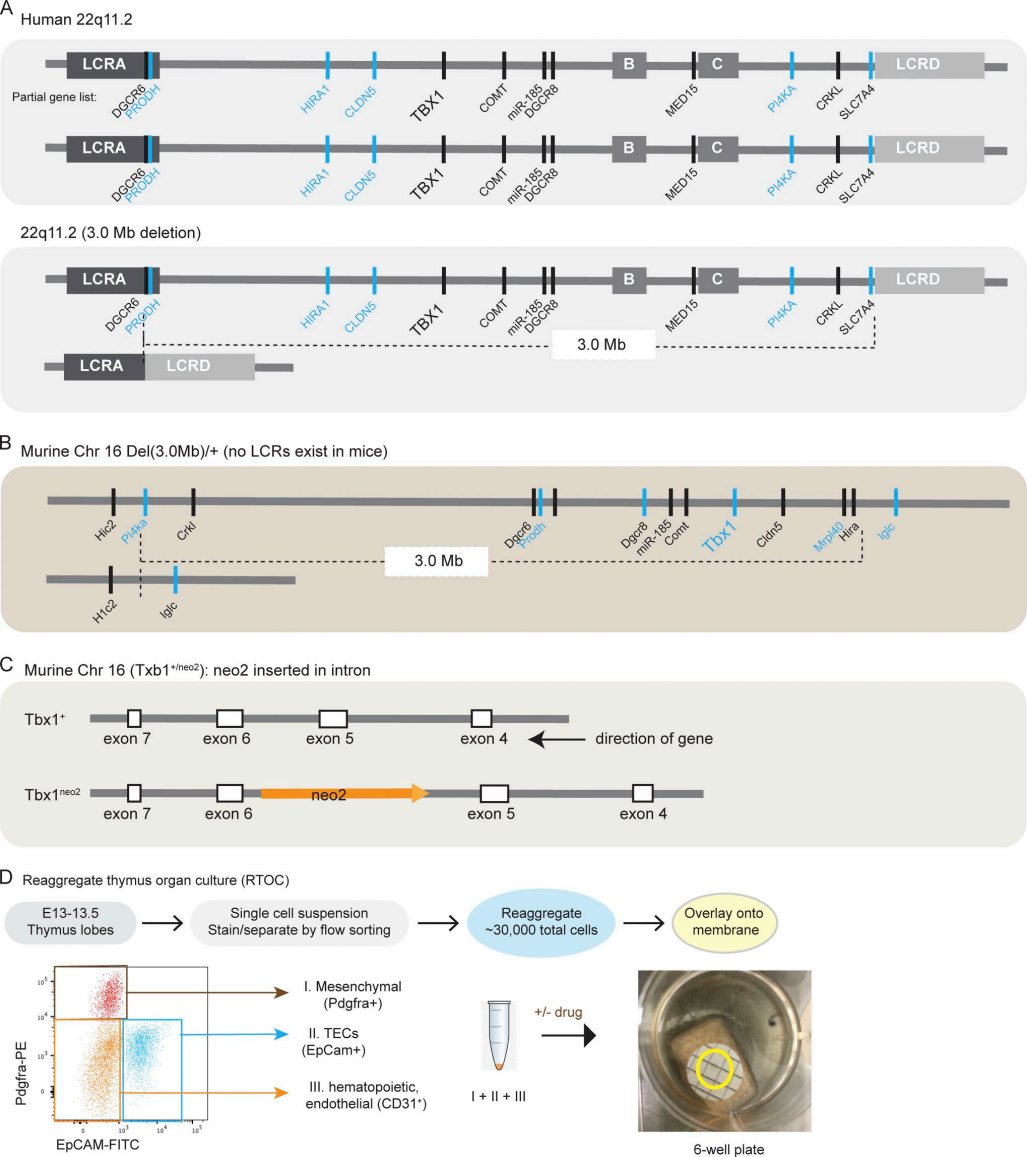
**Human 22q11.2 locus and corresponding mouse models that have overlapping congenital features along with RTOC procedures. (A)** Cartoon diagram illustrating the chromosomal organization of human 22q11.2DS along with several key genes interspersed among the first four of eight LCRs. One key gene is TBX1. Most patients with 22q11.2DS have one allele containing deletions between LCR A–LCR D, which spans ∼3 Mb. TBX1 becomes haploinsufficient. **(B)** An orthologous region exists on murine chromosome 16, with the conspicuous absence of LCRs. CRISPR/Cas9 was used to create a deletion on chromosome 16, matching the 3-Mb deletion in humans. **(C)** A distinct 22q.11.2DS mouse model with a highly penetrant thymic hypoplasia is the Tbx1^neo2/neo2^ line. In this line, the neomycin gene in inserted in an inverse reading frame within intron 5 of Tbx1. Tbx1^+/neo2^ adult mice are bred, yielding embryos with Tbx1^+/+^, Tbx1^+/neo2^, and Tbx1^neo2/neo2^ alleles. **(D)** Depiction of the RTOC assay modified to include flow sorted cells. Single-cell suspensions from E13–13.5 fetal thymic lobes were prepared, and mesenchymal cells (Pdgfra^+^), TECs (EpCam^+^), and the remaining unstained cells (Pdgfra^−^EpCAM^−^, which includes ETPs, other hematopoietic cells, and endothelial cells) are sorted by flow cytometry. The three subgroups were reaggregated at cell ratios established with control fetal thymuses and placed onto membranes and cultured. A minimum of 20,000 cells/aggregate is needed to sustain RTOC growth with normal cells. The aggregates appear as a small dot in the yellow circled area of a single well of a 6-well tissue culture plate.

**Figure 1.**
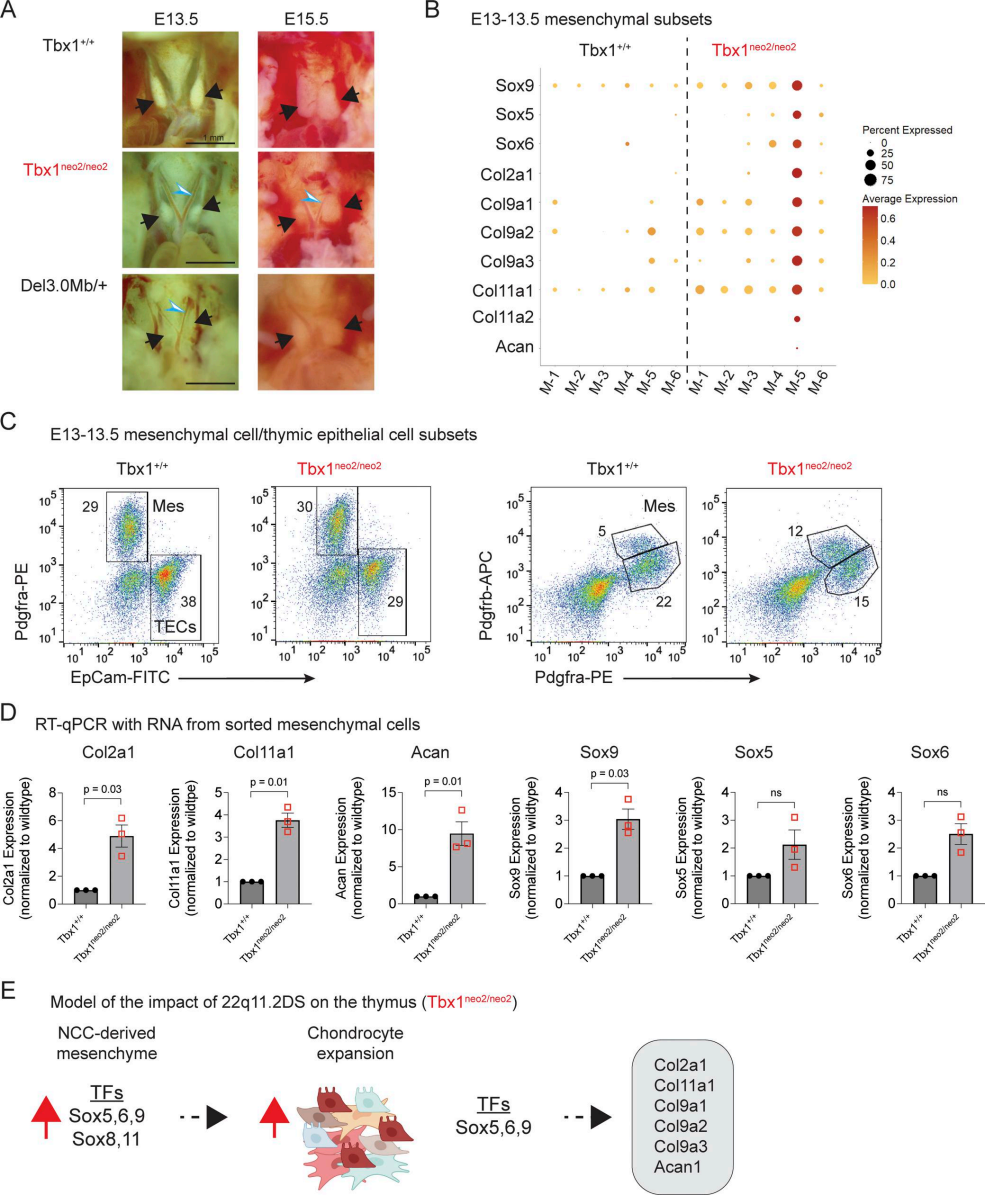
**Hypoplastic thymuses from an embryonic 22q11.2 mouse model have an overrepresentation of chondrocyte markers. (A)** The cardiothoracic regions are shown for E13–13.5 Tbx1^+/+^, Tbx1^neo2/neo2^, and Del(3/.0 Mb)/+ embryos. Black arrows point to the thymic lobes. An interrupted aortic arch in the Tbx1^neo2/neo2^ and Del(3/.0 Mb)/+ embryos is shown with an open blue triangle. **(B)** scRNA Seq data were used to compare Sox family TFs and target collagens in six mesenchymal subsets characterized in E13–13.5 Tbx1^+/+^ and Tbx1^neo2/neo2^ embryos. **(C)** Single-cell suspensions from E13–13.5 fetal thymic lobes were analyzed by flow cytometry to monitor mesenchymal (Pdgfra) and TEC (EpCam) percentages. The cells were also analyzed for the cell surface expression of Pdgfra and Pdgfrb. **(D)** E13–13.5 thymic mesenchymal cells sorted from the indicated embryos were processed for RT-qPCR to determine the expression levels of the indicated collagens, ECM transcript, and the Sox5,6,9 trio. Statistical significance was determined using three independent flow sorting experiments and performing a Student’s *T* test. **(E)** A model of the developmental changes in mesenchymal subsets is presented based on the upregulation of multiple Sox family members and target genes, collagens, and the ECM gene Acan. Mes, mesenchymal cells.

Thymic hypoplasia in 22q11.2DS results from deletion of either 3.0 or internal 1.5 Mb on chromosome 22 (Fig. S1 A). In humans, thymic hypoplasia is variable in penetrance and even less penetrant in mouse models with an orthologous deletion on chromosome 16 (Del(3.0 Mb)/+ [Fig. S1 B]) (34). Given the low frequency of thymic hypoplasia, we analyzed embryos using the Tbx1^neo2/neo2^ model of 22q11.2DS (Fig. S1 C and Table S1) (35). In this model, a neomycin cassette lies within the fifth intron of *Tbx1*, resulting in wild-type *Tbx1* expression at ∼35% control levels (Table S1) (35). Tbx1^neo2/neo2^ embryos have a penetrant thymic hypoplasia along with hypoparathyroidism and aortic arch defects (32, 35). Comparing the different mouse lines, thymuses from embryonic days (E)13–13.5 and E15–15.5 Tbx1^neo2/neo2^ and Del(3.0 Mb)/+ embryos were smaller and more rounded than controls (Fig. 1 A). We previously reported that hypoplastic thymuses from E13–13.5 embryos had elevated levels of collagens and ECM proteins (32). We analyzed the scRNA Seq data from our earlier studies to define a connection between the types of collagens upregulated in the six different mesenchymal subclusters and the corresponding TFs regulating these pathways. This revealed a specific upregulation of the trio of Sox5, Sox6, and Sox9 TFs that directly activate the collagens Col2a1, Col9a1, Col9a2, Col9a3, and Col11a1 along with Acan (Fig. 1 B) (36, 37, 38). At E13–13.5, the mesenchymal cells represent about 30% of the total cells in an E13–13.5 embryonic thymus (Fig. 1 C). To confirm the transcriptome findings, the mesenchymal cells were flow sorted, RNA extracted, and used for quantitative RT-PCR (RT-qPCR). Sox9 and several of the collagens were significantly elevated in the Tbx1^neo2/neo2^ mesenchymal cells (Fig. 1 D). The data suggested the embryonic NCC-derived mesenchymal cells from hypoplastic thymuses had an upregulation of specific Sox family TFs, causing increased collagen and ECM deposition (Fig. 1 E). Higher collagen levels were also evident in postnatal human 22q11.2DS thymuses (32).

### Restoration of growth for hypoplastic embryonic thymuses achieved with minoxidil and PGE_2_

**Figure S2.**
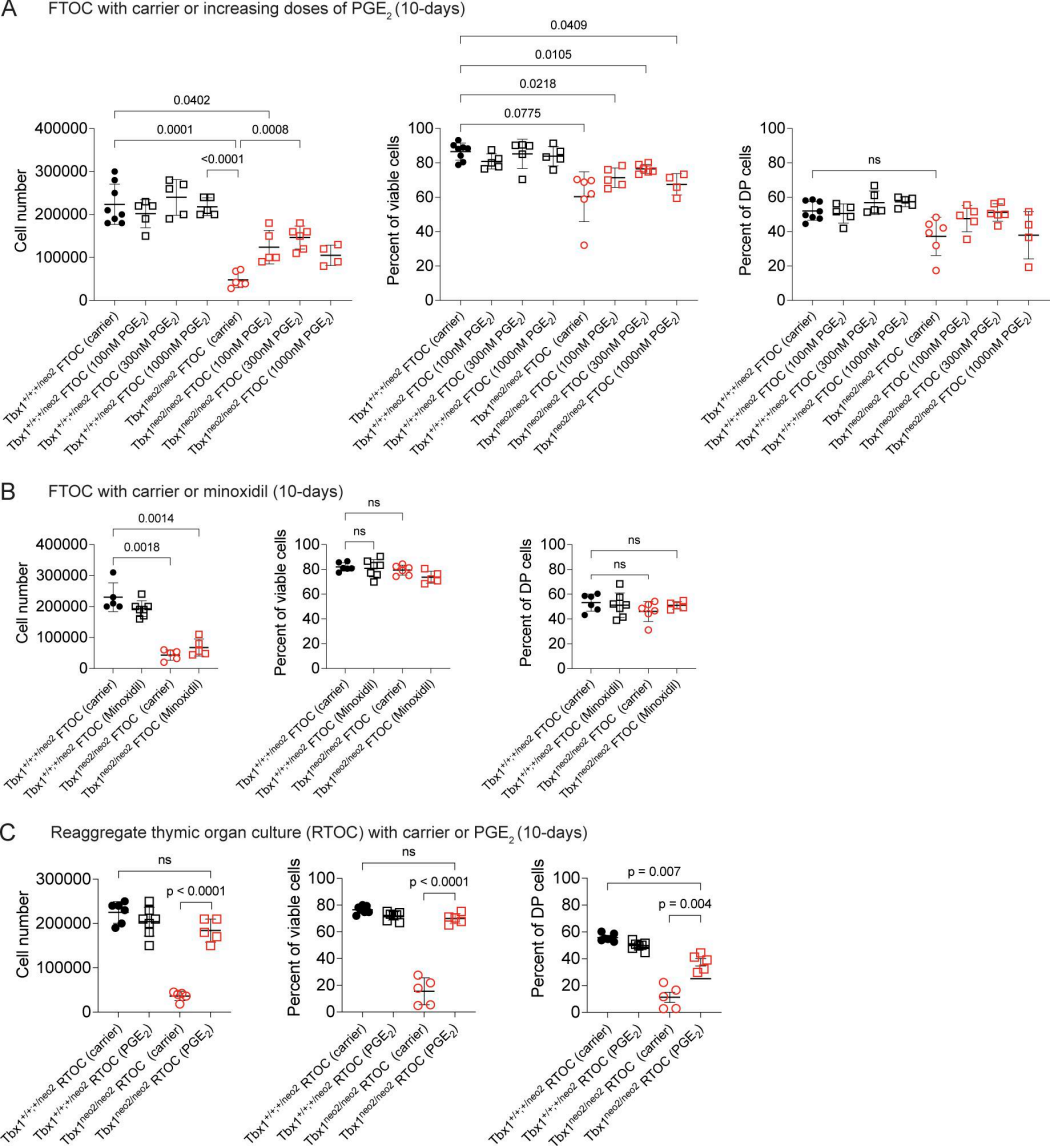
**Differential effects of PGE**_**2**_ **versus minoxidil in FTOCs and RTOCs. (A)** FTOCs were prepared with E13–13.5 embryos from the indicated genotypes of embryos. Individual paired thymic lobes were cultured in the absence or presence of increasing amounts of PGE_2_ (100, 300, and 1,000 nM). The black colored symbols represent control (Tbx1^+/+;+/neo2^) and red Tbx1^neo2/neo2^ genotypes. After 10-day cultures, cells were harvested and enumerated. Cell viability was determined by flow cytometry with electronic gaiting. The percent of DP thymocytes was determined following CD4 and CD8 co-receptor staining followed by flow analysis. The number of mice used in each of the eight groups were *n* = 8, 5, 5, 5, 6, 5, 6, and 4, respectively. Statistical analyses were done with one-way ANOVA (Brown–Forsythe and Welch tests). Only statistically significant differences are presented. **(B)** FTOCs were established as in A in the presence of a single dose of minoxidil. After 10 days, the cellularity, cell viability, and percent of DP cells determined as in A. This was done with an *n* = 5, 6, 5, and 6 embryonic thymuses per group. **(C)** RTOCs were prepared using single-cell suspensions from thymic lobes isolated from either control Tbx1^+/+;+/neo2^ or Tbx1^neo2/neo2^-genotyped embryos. Identical numbers of cells (∼30,000) were cultured in media alone or that supplemented with 300 nM PGE_2_. The number of experiments per group were *n* = 5, 6, 5, and 5. After 10 days, the total number of cells that grew was counted, and the cell viability and DP percentages were calculated following flow cytometry. Statistical analyses were done with one-way ANOVA (Brown–Forsythe and Welch tests).

**Figure 2.**
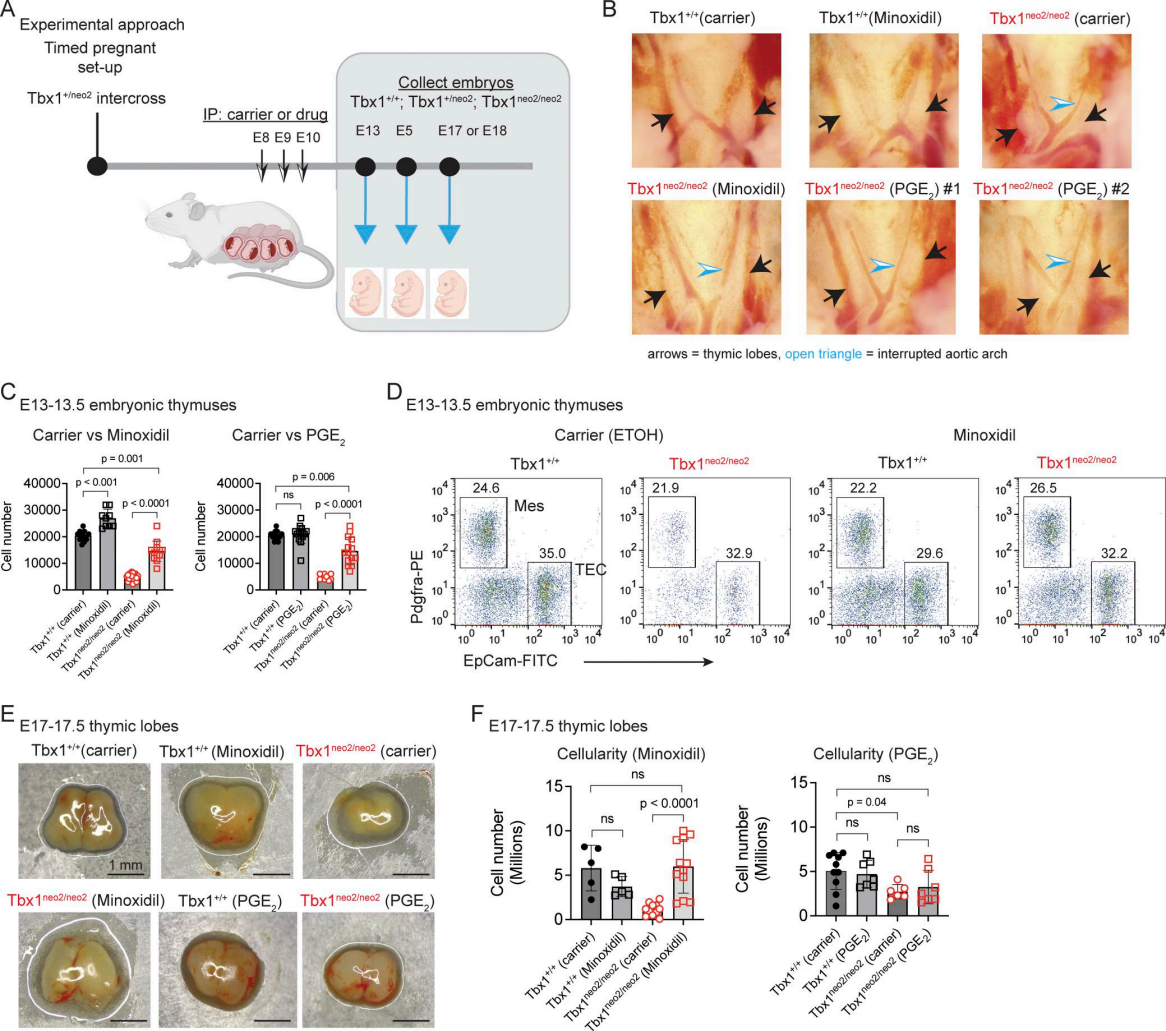
**Minoxidil and PGE**_**2**_ **treatments in pregnant mice restore growth for embryonic hypoplastic thymic lobes. (A)** Experimental strategy for administering carrier or drugs to pregnant mice from Tbx1^+/neo2^ intercrosses at the indicated time points (E8, E9, and E10). Embryos were isolated 3, 5, 7, and 8 days later. **(B)** The cardiothoracic regions are shown for E13–13.5 Tbx1^+/+^ and Tbx1^neo2/neo2^ embryos, obtained from pregnant mice that had received carrier, minoxidil, and PGE_2_ injections at E8, E9, and E10. Black arrows point to the thymic lobes. An interrupted aortic arch common to the Tbx1^neo2/neo2^ embryos is shown with an open blue triangle. **(C–F)** Single-cell suspensions from E13–13.5 fetal thymic lobes were prepared and (C) enumerated. Tbx1^+/+^-carrier (*n* = 11), Tbx1^+/+^-minoxidil (*n* = 9), Tbx1^neo2/neo2^-carrier (*n* = 20), and Tbx1^neo2/neo2^-minoxidil (*n* = 13) embryos were used. **(D)** Cells from the carrier and minoxidil-treated groups were stained with antibodies specific for mesenchymal cells (Mes; Pdgfra^+^) and TECs (EpCam^+^) and analyzed by flow cytometry. **(E)** Pictures of thymic lobes isolated from E17–17.5 embryos are shown for carrier, minoxidil, and PGE_2_ treatment groups. **(F)** The thymic cellularity was determined in the various treatment groups using E17–17.5 embryonic thymuses. Tbx1^+/+^-carrier (*n* = 5 or 12), Tbx1^+/+^-minoxidil (*n* = 7), Tbx1^neo2/neo2^-carrier (*n* = 9 or 6), Tbx1^neo2/neo2^-minoxidil (*n* = 12), and Tbx1^neo2/neo2^-PGE_2_ (*n* = 7). Statistical analyses for C and F were performed with one-way ANOVA (Brown–Forsythe and Welch tests).

In our previous work using in vitro FTOCs and reaggregate organ cultures (RTOCs), the addition of the drug minoxidil, which blocks collagen synthesis, restored growth for Tbx1^neo2/neo2^ thymuses in culture (32). One of the known effects of minoxidil is to increase PGE_2_ levels (39, 40, 41, 42, 43, 44, 45, 46). To test whether PGE_2_ could improve thymus growth, a dose response analysis was done in FTOCs and RTOCs (Fig. S1 D). PGE_2_ enhanced thymic growth in both FTOCs and RTOCs compared with carrier treatments (Fig. S2, A–C) (44). As with minoxidil, PGE_2_ improved the overall cell viability from 20% to 80% along with a normal differentiation of early thymus progenitors (ETPs) to the different thymocyte subsets (Fig. 2 C). Unlike PGE_2_, minoxidil did not improve thymus growth in FTOCs (Fig. S2 B). This suggested that dissolution of the thymic capsule was required for minoxidil to exert a growth benefit.

**Figure S3.**
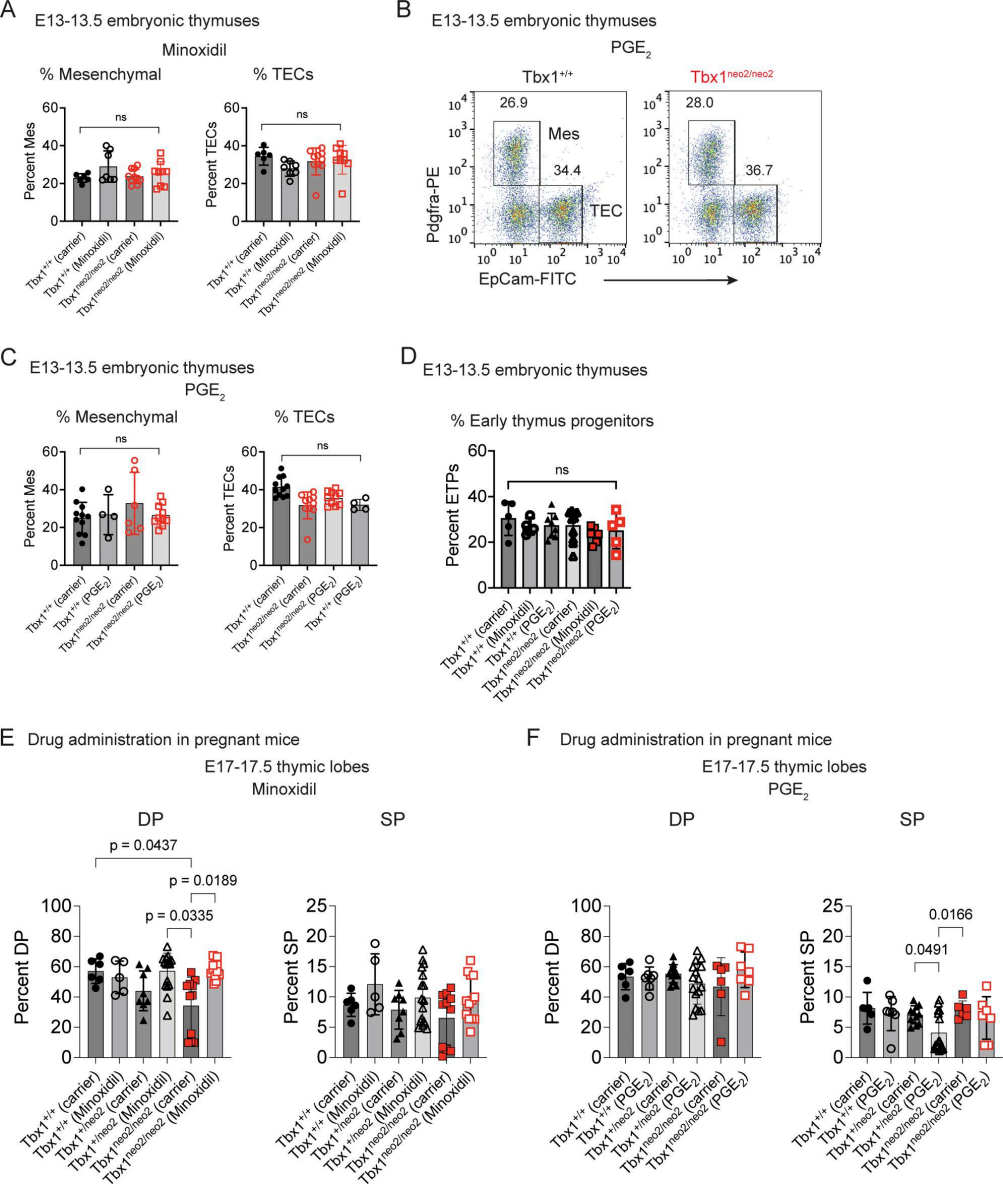
**Minoxidil and PGE**_**2**_ **have beneficial growth effects in vivo on developing thymuses. (A–D)** Timed pregnant mice were obtained from Tbx1^+/neo2^ intercrosses and injected with carrier, minoxidil, or PGE_2_ at E8, E9, and E10. Fetal thymic lobes were isolated from embryos at E13–13.5 and processed for flow cytometry to determine the percentage of mesenchymal cells (Pdgfra^+^) and TECs (EpCAM^+^). **(A)** Minoxidil had no statistically significant difference on mesenchymal or TEC cell percentages. **(B and C)** Flow cytometric analyses were used to compare the mesenchymal (Mes) and TEC subset percentages after PGE_2_ treatments. The absolute cell numbers are presented. **(D)** The percent of ETPs was compared among embryos from all the carrier or drug treatment groups. **(E and F)** Timed pregnant mice were obtained from Tbx1^+/neo2^ intercrosses and injected with carrier, minoxidil, or PGE_2_ at E8, E9, and E10. Fetal thymic lobes were isolated from embryos at E17–17.5. Single-cell suspensions were prepared. The percent of DP and SP thymocytes was determined following CD4 and CD8 co-receptor staining, followed by flow analysis to detect co-expression of CD4 and CD8 or unique expression of just CD4 or CD8, representing SP cells. These were presented in the minoxidil (E) and PGE_2_ (F) groups. Statistical analyses were done with one-way ANOVA (Brown–Forsythe and Welch tests). P values P < 0.05 are shown. Graphs without P values had no statistically significant differences.

To test if minoxidil and/or PGE_2_ could work in vivo, timed pregnant mice using Tbx1^+/neo2^ heterozygous intercrosses were injected intraperitoneally (IP) with either a carrier control (ETOH), minoxidil, or PGE_2_ at embryonic days E8, E9, and E10 (Fig. 2 A). This time course preceded the specification of the thymus and Pth’s within the third pharyngeal pouch (47). Minoxidil administration restored the expansion of the thymic tissue from Tbx1^neo2/neo2^ embryos (Fig. 2, B and C). Treated Tbx1^neo2/neo2^ thymuses had a 3.3-fold increased cell number versus those obtained from the carrier control (Fig. 1 C). Minoxidil treatment also led to a smaller 1.3-fold increase in the thymic cellularity in the controls (Tbx1^+/+^) (Fig. 2 C). PGE_2_ administration similarly resulted in a 3.2-fold increase in cellularity in thymuses from Tbx1^neo2/neo2^ embryos (Fig. 2, B and C). Given the improved tissue growth, we next assessed whether there was a change in composition of thymic mesenchymal cells (Pdgfra^+^) and thymic epithelial cells (TECs) (EpCAM^+^). At E13–13.5, the percentages of these subsets remained like controls following either minoxidil or PGE_2_ treatments (Fig. 2 D and Fig. S3, A–D) (32). The percent of ETPs (CD117^+^) was similar among all groups compared (Fig. S3 D).

To characterize the impact of the drug treatments on T cell development, thymuses of E17–17.5 embryos were compared. Thymic lobes from Tbx1^neo2/neo2^ embryos were visibly smaller than wild-type controls (Tbx1^+/+^) (Fig. 2, E and F). Minoxidil administration in utero significantly increased the size for the Tbx1^neo2/neo2^ thymuses (Fig. 2 E). The effect of PGE_2_ was more variable and did not meet statistical significance (Fig. 2, E and F). Thymocytes progress from a double-negative (DN; CD4^−^CD8^−^) to double-positive (DP; CD4^+^CD8^+^), followed by single-positive subsets (SP; mainly CD4^−^CD8^+^ in early embryos) as they mature (48). DP thymocyte percentages were similar in embryos from all the genotypes (Tbx1^+/+^, Tbx1^+/neo2^, and Tbx1^neo2/neo2^) following the drug treatments (Fig. S3, E and F). This is consistent with 22q11.2DS primarily impacting the size of the thymus, with T cell development reportedly normal (23, 32, 49). In summary, both minoxidil and PGE_2_ administration to pregnant mice re-established growth for 22q11.2DS (Tbx1^neo2/neo2^) embryonic thymuses.

### Drugs that reduce collagen production and cross-linking enable hypoplastic thymuses from 22q11.2DS mouse models to expand

Minoxidil and PGE_2_ reduce the expression of collagens and lysyl hydroxylases, which limit collagen and elastin cross-linking (32, 39, 40, 42, 43, 44, 45, 46). These drugs also function as vasodilators, improve angiogenesis, and increase release of various growth factors, with PGE_2_ also blocking adrenergic receptor signaling (43, 50, 51, 52, 53). To determine which drug-sensitive pathway(s) contributed to thymic tissue growth, other drugs that selectively affect vasodilation, calcium-regulated endothelial permeability, and/or adrenergic receptor signaling were tested. This was done in RTOCs, FTOCs, and/or administration to the pregnant mice (Table 1). Restoration of thymic growth was compared with carrier and minoxidil or PGE_2_ containing RTOCs/FTOCs and in vivo treatments (Table 1). Beta-propionitrile, which inhibits collagen production and increases vasodilation, did not enable FTOC or RTOC growth (Table 1) (54, 55). Two vasodilators, enalaprilat and hydralazine, were also ineffective at restoring growth in RTOCs (56, 57). Two other vasodilators used in clinical settings, enalapril maleate and hydralazine, were ineffective at restoring thymic growth in vivo when given to the pregnant mice (56). Finally, labetalol and nifedipine, drugs that open calcium channels and antagonize adrenergic receptor pathways, respectively, were ineffective and/or exhibited some toxicity in the RTOCs and/or FTOC assays (Table 1) (58, 59). In summary, only minoxidil and PGE_2_ consistently increased thymic tissue growth for hypoplastic lobes in vitro and in vivo. These findings provide evidence that increased collagen production and ECM deposition are the principal causes of the small thymus phenotype in 22q11.2DS embryos.

**Table 1.** Comparative drug treatment impacts on Tbx1^neo2/neo2^ hypoplastic thymic growth

Drugs		Tbx1^neo2/neo2^ thymus growth in culture	Tbx1^neo2/neo2^ hypoplastic thymus growth following drug administration in pregnant mice			
Name (s)	Mechanism(s) of action	FTOC	E13–13.5 cell number^a^	E13–13.5 Mes/TEC ratio^b^	E17–17.5 cellularity^a^	E17–17.5 % DP/SP thymocytes^a^
		RTOC				
Carrier (ETOH)	None	FTOC: −	+	Normal	+	++++
		RTOC: −				
Minoxidil	Blocks collagen production/cross-linking, vasodilator, stimulates PGE_2_, and growth factors	FTOC: -	++++	No change	++++	++++
		**RTOC**: ++++^c^				
PGE_2_	Blocks collagen production, vasodilator, stimulates VEGF, and bFGF production	FTOC: ++	++++	No change	No effect	+++
		**RTOC**: ++++				
BAPN	Lysyl oxidase inhibitor that blocks collagen cross-linking	FTOC: −	ND^e^	ND	ND	NA^f^
		RTOC: −				
Enalaprilat (in vitro)	Vasodilator by inhibiting ACE^d^	FTOC: +	+	No change	ND	ND
		**RTOC**: ++				
Enalapril (in vivo)	Vasodilator by inhibiting ACE^d^	NA	+	No change	ND	ND
Hydralazine	Vasodilator inhibits smooth muscle Ca^2+^ release	FTOC: −	NA	NA	NA	NA
		RTOC: −				
Labetalol	α/β adrenergic receptor inhibitor	FTOC: +	ND	ND	ND	ND
		**RTOC**: ++				
Nifedipine	Calcium channel blocker	FTOC: +	NA	NA	NA	NA
		RTOC: −				

bFGF, basic Fibroblast growth factor.

a Cell number − = 0–10% of wild-type controls; ++ = 50–80% of wild-type controls; +++ = 60–80% wild-type controls; ++++ = 80–100% of wild-type controls.

b Mesenchymal/TEC ratio.

c Bold = growth restored.

d ACE = angiotensin-converting enzyme.

e ND = not done.

f NA = not applicable.

### Normalization of mesenchymal and endothelial transcriptomes in Tbx1^neo2/neo2^ embryos following minoxidil and PGE_2_ drug treatments in utero

**Figure 3.**
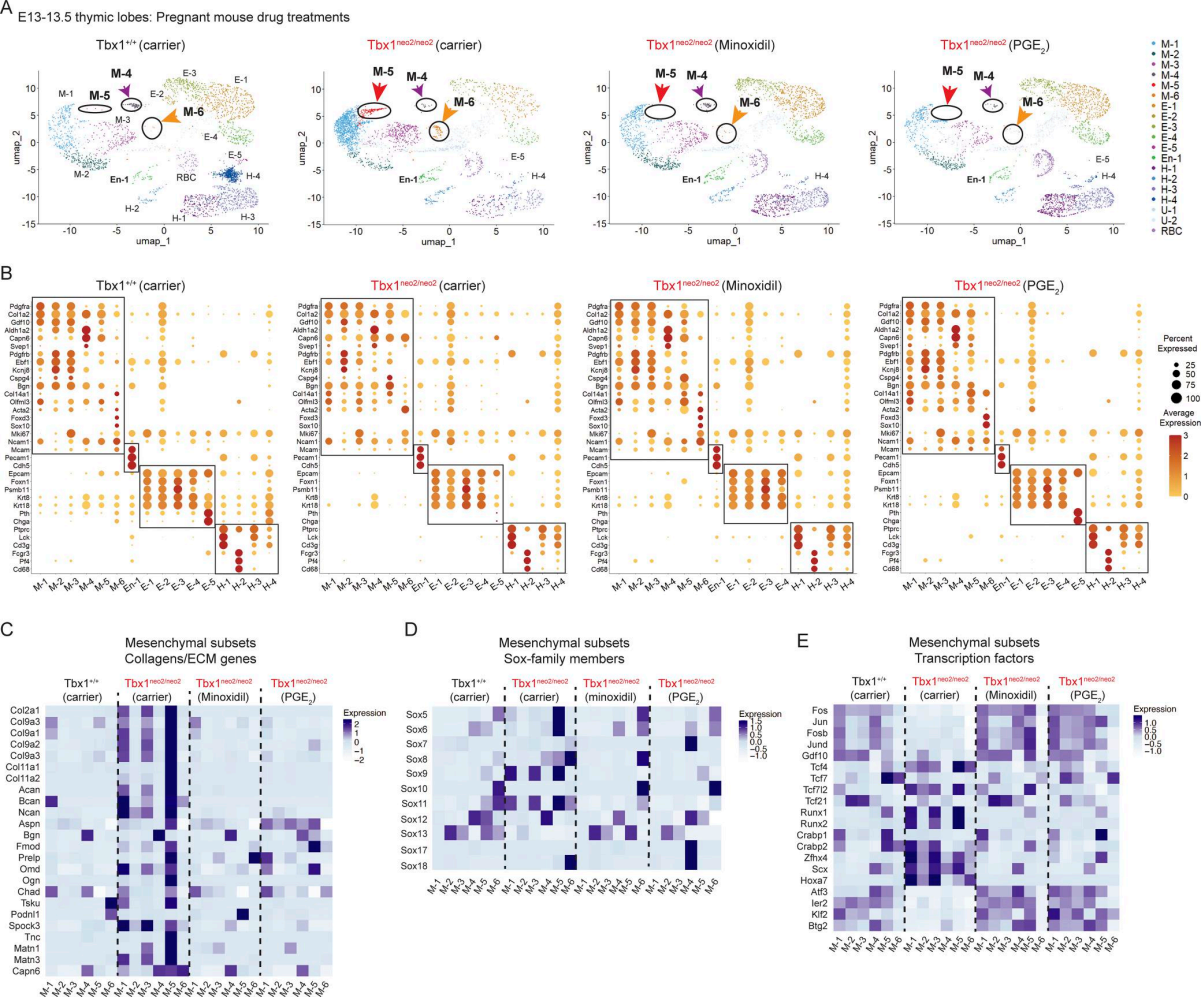
**scRNA Seq reveals transcript levels in mesenchymal, endothelial cells, epithelial, and hematopoietic subsets in embryonic thymuses after in utero carrier and drug treatments. (A)** Fetal thymuses were isolated from Tbx1^+/+^ and Tbx1^neo2/neo2^ E13–13.5 embryos obtained from carrier, minoxidil-, and PGE_2_-treated pregnant mice. scRNA Seq results were plotted with UMAPs, revealing 18 distinct cell subgroups (Tbx1^+/+^-carrier, Tbx1^neo2/neo2^-carrier, -minoxidil, and -PGE_2_). Six mesenchymal cell clusters (M-1 to M-6), one endothelial population (En-1), five epithelial clusters (E-1 to E-5), four hematopoietic cell types (H-1 to H-4), and a red blood cell cluster (U-1) are shown. The red and orange arrows indicate the overrepresentation of M-5 and M-6 clusters in hypoplastic thymuses (Tbx1^neo2/neo2^). The purple reveals changes in the M-4 cluster. **(B)** Dot plot comparisons reveal transcript levels in the various subclusters. Increasing dot sizes reveals the percentage of cell expression the transcripts. The orange to red scale reveals increasing expression levels. Mesenchymal (M-1 to M-6), endothelial (En-1), epithelial (E-1 to E-5), and hematopoietic (H-1 to H-4) superclusters are boxed. **(C–E)** Heat maps reveal the relative expression of the indicated mesenchymal transcripts grouped under three broad categories: (C) collagens/ECM proteins, (D) Sox family TFs, and (E) additional transcriptional regulators. Genes with P < 0.00001 and logbase_2_ differences >1.5 are shown.

At E13–13.5, mesenchymal, TECs, and endothelial populations represent ∼25–30%, ∼25–30%, and 1–2% of the cells in the developing thymus, respectively (32). Our prior scRNA Seq revealed that Tbx1^neo2/neo2^ embryonic thymuses had significant transcript alterations in the mesenchymal subclusters and single endothelial cluster (32). To determine the impact of minoxidil and PGE_2_ on stromal and hematopoietic subclusters after administering the drugs in pregnant mice, scRNA Seq was undertaken. E13–13.5 embryonic thymuses were isolated from Tbx1^+/neo2^-intercrossed pregnant dams that had received carrier (ETOH), minoxidil, or PGE_2_ injections at E8, E9, and E10 (Fig. 2 A). Between 3,362 and 11,908 cells (pooled lobes for hypoplastic tissues) were used, with an average read count of 130,312/cell found (Table S2). Unsupervised hierarchical clustering indicated the presence of 18 clusters in 4 groups, defined using mesenchymal (Pdgfra/Col1a2), endothelial (Cdh5/Pecam), epithelial (EpCAM/Krt8), and hematopoietic supercluster (Ptprc) markers, along with one mitochondrial group and an unidentified cluster (Table 2). Hypoplastic lobes (Tbx1^neo2/neo2^) had altered mesenchymal clusters, minor changes in epithelial clusters, and reduced cell numbers in the hematopoietic cluster (Fig. 3 A and Table 2).

**Table 2.** Embryonic thymic subcluster representation following carrier or drug administration

Cell subclusters	Number and percent of cells/sample				Putative cell type (Identifier genes)
	Tbx1^+/+^ (carrier) (11,820)	Tbx1^neo2/neo2^ (carrier) (11,123)	Tbx1^neo2/neo2^ (minoxidil) (3,293)	Tbx1^neo2/neo2^ (PGE_2_) (4,290)	
Mesenchymal	2,820	6,400	998	882	Mesenchymal subcluster (genes)
M-1	1,048 (37%)	**3,748 (59%)** ^a^	426 (43%)	396 (44%)	Caps Fb 2b (Adamts2, Bgn, and Fbn1) and 3 (Igf1, Dcn, and Olfml3)
M-2	797 (28%)	*432 *(*7%*)^b^	244 (24%)	229 (25%)	Pericytes-A (Ebf1, Kcnj8, Pdgfrb, and Cspg4)—high
M-3	671 (24%)	1,304 (20%)	253 (25%)	219 (26%)	Pericytes-B (Ebf1, Kcnj8, Pdgfrb, and Cspg4)—low. Proliferating cells (Mki67 and Top2a1)
M-4	247 (9%)	*59 *(*1%*)^b^	61 (6%)	22 (2.5%)	Cap Fb 1 (Adh1a2, Capn6, Itm2a, and Svep1)
M-5	15 (0.5%)	**583 (9%)** ^a^	2 (0.2%)	3 (0.3%)	Cap Fb (Sox 9) In Tbx1^neo2/neo2^ thymuses: Chondrocytes (Sox 5,6,9, Col2a1 Col9a1, Col11a1, and Acan)
M-6	42 (1.5%)	**274 (4%)**	12 (1.2%)	13 (1.5%)	Thymic mesoderm-derived mesenchymal subset with transient myogenic potential (Chrna1, Pax7, Myod1, Myog, Pdgfra, Vcam1, and Col1a2) and some neural crest-derived cells (Sox10 and Foxd3)
Endothelial	216	261	109	104	Endothelial (Cdh1 and Pecam1)
Epithelial	3,666	1,354	1,004	1,318	Epithelial subcluster (genes)
E-1	1,692 (46%)	660 (49%)	526 (52%)	685 (52%)	cTEC^lo^ I (EpCAM, Krt8, low levels of Psmb11, Prss16, and Ccl25)
E-2	179 (5%)	36 (3%)	18 (2%)	19 (1%)	cTEC ^lo^ II (EpCAM, Krt8, low levels of Psmb11, Prss16, and Ccl25)
E-3	1,032 (28%)	390 (29%)	267 (27%)	343 (26%)	cTEC^hi^ (EpCAM, Krt8, Psmb11, Prss16, and Ccl25)
E-4	662 (18%)	260 (19%)	193 (19%)	270 (20%)	Bipotent thymic epithelial progenitor population (EpCAM, Krt8, Krt14, Krt5, Krtdap, Cldn4, lower levels of MHC class II, and Ccl21)
E-5	101 (3%)	8 (0.6%)	0 (0%)^c^	1 (0.8%)	Immature TECs; Pth marker (EpCAM, Krt8, Pth, and Chga)
Hematopoietic	4,480	1,308	796	1,275	Hematopoietic subclusters
H-1	842 (19%)	381 (24%)	351 (44%)	637 (50%)	DN3 (high levels Ptprc, Lck, Cd3g, Runx1, and Il2ra)
H-2	197 (4%)	**314 (24%)** ^a^	96 (12%)	76 (6%)	Macrophages and dendritic cells (Pf4, CD68, Fcgr3, and C1qa)
H-3	1,224 (27%)	431 (29%)	301 (38%)	486 (38%)	Early TP and DN2 (Cd7, Cd25, Il2ra, and CD44)
H-4	2,217 (49%)	*182 (14%)*	48 (6%)	76 (6%)	DN4/innate-like cytotoxic T/NK precursors (Ifitm1, Ifitm2, Gzma, and Plac8)

a Uniquely elevated in Tbx1^neo2/neo2^ carrier (bold).

b Uniquely reduced in Tbx1^neo2/neo2^ carrier (italic).

c Absent, likely corresponds to some Pth material.

To define the changes in each cell subtype, subclustering was done based on a defined set of transcripts (Table 2). Note the current numbering is distinct from that previously assigned (32). Mesenchymal cells comprised six subclusters: capsular fibroblasts (Cap Fb’s 1, 2, 3 and 4; identifier = M-1, M-4, and M-5), perivascular cells or pericytes (comprises mural and smooth muscle cells) (identifier = M-2 and M-3), and cells with chondrogenic markers (identifier = M-5 and some M-6) (Fig. 3 B and Table 2). Mesenchymal subsets M-5 and M-6 were selectively overrepresented 17- and 3-fold, respectively, in the Tbx1^neo2/neo2^ hypoplastic thymuses (carrier treatment group) (Fig. 3 A, see arrows). M-1 subcluster (Cap Fb’s 2 and 3) was marginally over-represented (1.5-fold) (Fig. 3, A and B). M-2 and M-4 were marginally and substantially reduced, respectively (Fig. 3, A and B; and Table 2). Minoxidil and PGE_2_ normalized the representation of these subclusters, with stark reductions in the M-5 and M-6 subsets and a restoration of M-4 for the Tbx1^neo2/neo2^ embryos (Fig. 3, A and B). Most TEC subclusters were comparable between the wild-type (Tbx1^+/+^), Tbx1^neo2/neo2^ carrier controls, and Tbx1^neo2/neo2^ drug treatment groups. One exception was subcluster E-5, absent in the minoxidil-treated group (Fig. 3 A). As this subcluster contains Pth markers, minoxidil may have caused the Pth tissue to completely separate from the thymus more effectively than the control and PGE_2_ samples (Table 2). The hematopoietic subclusters in the hypoplastic thymuses (Tbx1^neo2/neo2^-carrier) exhibited more cells in H-2, while H-3 and H-4 were reduced (Fig. 3 A). Minoxidil and PGE_2_ normalized the representation of H-1, H-2, and H-3 subclusters, while H-4 remained low (Fig. 3, A and B; and Table 2).

### Minoxidil and PGE_2_ administration in pregnant mice normalized the levels of multiple Sox family members in the mesenchymal subsets

We next analyzed the differentially expressed genes (DEGs) seen among multiple NCC-derived mesenchymal subsets in control and Tbx1^neo2/neo2^ embryonic thymuses to identify the mechanistic cause(s) for the small thymus. Consistent with our previous findings, there was a significant increase in collagens and ECM transcript expression in the mesenchymal subclusters from the hypoplastic thymuses (Tbx1^neo2/neo2^) (Fig. 3 C). The in vivo administration of minoxidil and PGE_2_ to the pregnant mice resulted in a dramatic reduction in the expression of these same transcripts in most of the mesenchymal subclusters affected (Fig. 3 C). Given the dramatic changes in gene expression, particularly the collagens, we assessed the changes in Sox family member TFs known to regulate these genes. Eight different Sox family members were affected, with five upregulated and three reduced in expression in one or more of the mesenchymal subclusters (Fig. 3 D). The M-5 subcluster had a prominent overexpression of the Sox5,6,9 trio (Fig. 3 D). This Sox family trio enforces the programming of mesenchymal cells into chondrocytes, which are major producers of collagens and ECM proteins (Fig. 3 D) (60). Sox11, Tcf12, Runx2, and Hoxa7 were also elevated. Sox11 increases ECM protein deposition, with a putative role in epithelial–mesenchymal interactions (61). Each of these TFs regulates various aspects of mesenchymal cell migration, proliferation, and/or differentiation (36, 37, 38). This would explain the expansion of chondrocytes. All six mesenchymal subclusters exhibited a substantial reduction in transcripts involved in the MAP kinase signaling pathway (e.g., Fos and Jun) in the Tbx1^neo2/neo2^ embryos (Fig. 3 E). Remarkably, the majority of the TFs normalized following in utero drug treatments, which restored the thymus size in the Tbx1^neo2/neo2^ embryos (Fig. 3, C–E and Fig. 2 B). Among those TFs that were reduced to control levels were the Sox5,6,9 trio and Sox11. These four TFs drive chondrogenic cell fate (60). Their reduced levels following minoxidil or PGE_2_ treatments were consistent with the diminished transcripts encoding proteins involved in collagen assembly, collagen cross-linking, and ECM proteins in the Tbx1^neo2/neo2^ thymuses (Fig. 3, C–E). In summary, a developmental reprogramming of embryonic mesenchymal cells in 22q11.2DS mouse models results in an overrepresentation of chondrocytes, reversed by minoxidil and PGE_2_ administration in vivo.

### Reducing Sox9-expressing chondrocytes in the Tbx1^neo2/neo2^ thymic capsule normalizes tissue growth

**Figure 4.**
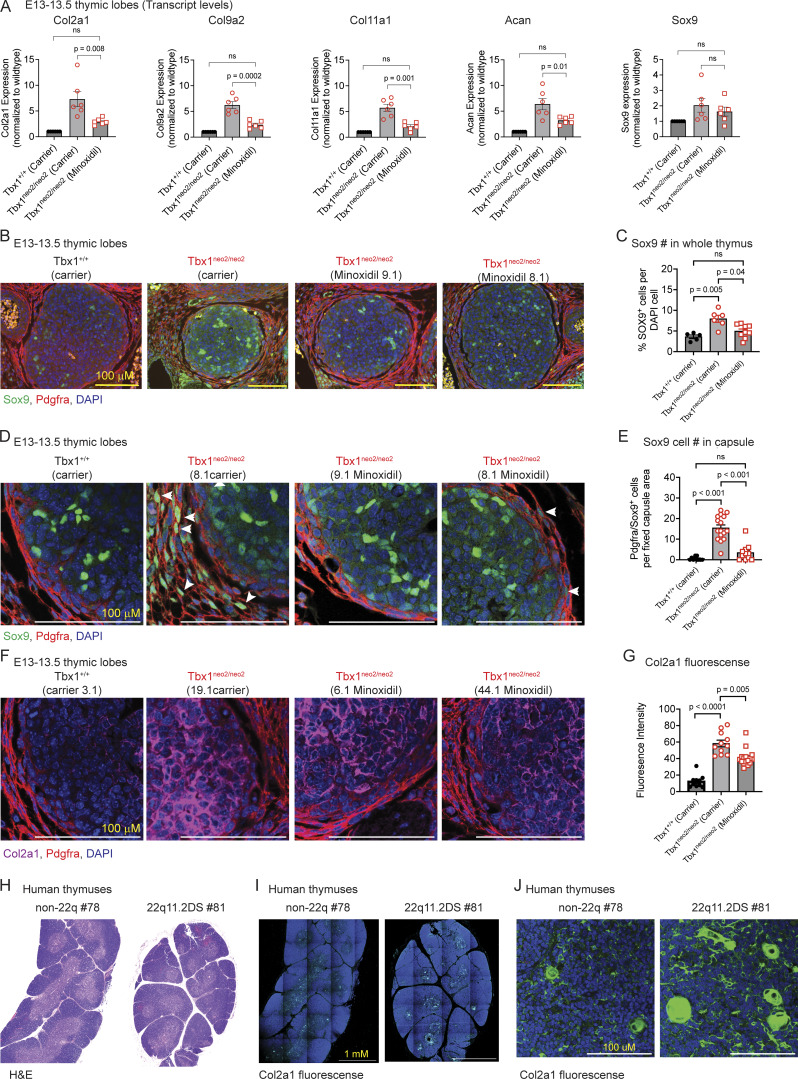
**Sox9-expressing mesenchymal cells accumulate in the capsular region of embryonic thymus from mouse models of 22q11.2DS. (A)** RT-qPCR was performed with RNA isolated from the indicated embryonic thymuses obtained from the carrier or minoxidil-treated pregnant dams. Probes specific for Col2a1, Col9a2, Col11a1, Acan, and Sox9 were used. **(B–D)** Fetal thymuses were isolated from Tbx1^+/+^ and Tbx1^neo2/neo2^ E13–13.5 embryos from carrier and minoxidil-treated pregnant mice and used for immunofluorescence. **(B)** Antibodies against Sox9 (green), Pdgfra (red), and a nuclear marker (DAPI, blue) were used to compare Sox9 expression in embryonic thymic lobes. Sections are representative of five to eight independent samples/genotype with individual colors provided per sample. Note that the samples in Fig. 4 B are duplicated in the Fig. S5 B for comparative purposes. **(C)** Quantitation of the number of Sox9-expressing cells per total DAPI-positive cells was determined using one-way ANOVA. The embryonic thymuses included Tbx1^+/+^-carrier (*n* = 5), Tbx1^neo2/neo2^-carrier (*n* = 6), and Tbx1^neo2/neo2^-minoxidil (*n* = 9) treatment groups. **(D)** Confocal microscopy was used to visualize the Sox9^+^ cells in the thymic capsule using the same sections as in B. Sections shown are representative of five to eight independent samples/genotype. **(E)** The number of Sox9^+^ cells in the capsular region was enumerated using a defined area of focus for all the immunohistochemistry (IHC) images. Statistical significance was determined as in C. **(F)** Antibodies against Col2a1(purple), along with Pdgfra (red) and a nuclear marker (DAPI, blue), were used to compare the levels of Col2a1. Sections are representative of five independent samples/genotype. **(G)** Quantitation of Col2a1 levels was performed by comparing the mean fluorescent intensity of a defined area among all the groups analyzed. Statistical analyses were done using one-way ANOVA. This was determined with thymuses from Tbx1^+/+^-carrier (*n* = 3), Tbx1^neo2/neo2^-carrier (*n* = 6), and Tbx1^neo2/neo2^-minoxidil (*n* = 5) groups. Statistically significant differences were established by one-way ANOVA (Brown–Forsythe and Welch tests). **(H)** Postnatal human thymuses from non-22q and 22q11.2DS patients were processed and visualized with H&E staining. **(I and J)** The human thymuses were also analyzed by immunofluorescence with an antibody specific for human COL2a1 along with a nuclear marker (DAPI, blue). **(J)** Confocal microscopy was used to visualize the exact location of the elevated COL2a1 expression. The different quadrants were stitched to create a composite image.

The mesenchymal subcluster changes in the Tbx1^neo2/neo2^ hypoplastic lobes were coupled with increased collagen and Sox9 transcript levels (Fig. 3, C–E). To confirm these findings, RT-qPCR was done on whole thymic tissues from E13–13.5 embryos isolated from carrier and drug-treated pregnant mice. Col2a1, Col9a2, Col11a1, and Acan were significantly increased in the Tbx1^neo2/neo2^ hypoplastic lobes (Fig. 4 A). Each was significantly reduced following minoxidil administration (Fig. 4 A). However, Sox9 levels did not change significantly following the drug treatment. One likely explanation for this is the growth and expansion of TEC subsets that express Sox9 (62, 63). Since the thymic capsule is primarily composed of mesenchymal cells, we assessed the number and location of Sox9^+^ cells by immunofluorescence (62). A small number of Sox9-expressing cells were detected throughout the control thymus (Fig. 4 B). Many more Sox9-positive cells were evident in the hypoplastic lobes (Tbx1^neo2/neo2^) (Fig. 4 B). Minoxidil administration to the pregnant dams, prior to thymus specification, reduced their numbers (Fig. 4 B, two examples). Quantification of the Sox9 cell number confirmed a statistically significant increased number of Sox9^**+**^ cells in the Tbx1^neo2/neo2^ thymuses (Fig. 4 C). Confocal microscopy was then used to visualize the location and intensity of the Sox9 signal. There was an overrepresentation of Sox9-expressing chondrocyte-fated cells within an expanded thymic capsule in the hypoplastic thymuses (Fig. 4 D). Minoxidil treatments significantly reduced the thickness of the thymic capsule, with a corresponding loss of Sox9^+^ chondrocytes (Fig. 4 E). Some Sox9^+^ cells remained within the center of the thymic lobe, likely representing some mesenchymal cells, endothelial cells, and/or TECs. As chondrocytes are the primary producers of specific collagens, such as Col2a1, these were compared among the embryonic thymuses (Fig. 4 F). Col2a1 levels were significantly elevated in the small thymuses (Fig. 4, F and G). These were partly reduced following minoxidil treatments. Our prior work established that human thymuses from 22q11.2DS patients had higher collagen levels than controls (32). To specifically characterize the levels of Sox9-regulated Col2a1, we undertook immunohistochemical and immunofluorescent comparisons with a 22q11.2 versus non-22q human thymus. The control human thymus had a well-defined lobular structure with thin septae and clear cortical-medullary zonation (Fig. 4 H). The 22q11.2DS thymus had thicker fibrous septae and poor cortical-medullary zonation. The human 22q11.2DS thymus had more separation between the subsections of the thymus. Immunofluorescence revealed higher Col2a1 expression in the 22q11.2DS thymus, predominantly medullary, exhibiting a denser, patchy, and punctate distribution. The control Col2a1 signal is minimal and diffusely localized, primarily within the medullary region (Fig. 4 I). This was more evident in a zoomed-in image, with these levels higher in Hassall’s corpuscules (Fig. 4 J). Taken together, our findings establish that Sox9-expressing chondrocyte-like cells are expanded in hypoplastic embryonic thymuses in the setting of 22q11.2DS. This contributes to increased collagen and ECM deposition, which is also revealed in postnatal adult thymic tissue from 22q11.2DS patients.

### Altered developmental trajectories of mesenchymal subsets in Tbx1^neo2/neo2^ thymuses impact the endothelial and TEC transcriptomes

**Figure 5.**
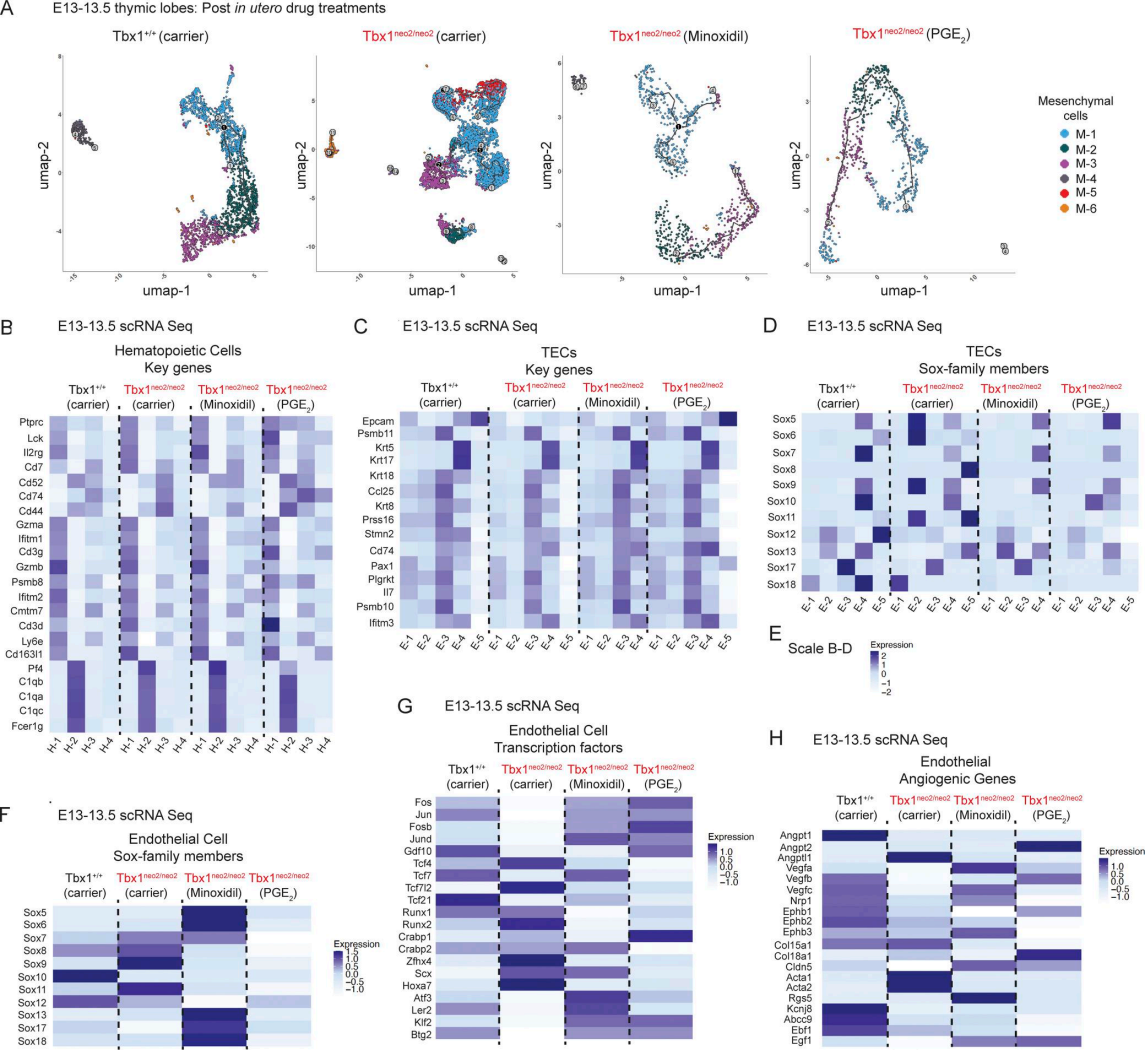
**Minoxidil and PGE**_**2**_ **treatments reprogram mesenchymal cell state trajectories and hematopoietic, epithelial, and endothelial transcriptional profiles in small thymuses. (A)** Cell state trajectory mapping of mesenchymal differentiation in E13–13.5 embryonic thymuses were compared after carrier and drug treatments in the pregnant mice. The mesenchymal cluster trajectories were compared in control Tbx1^+/+^-carrier and Tbx1^neo2/neo2^-carrier, -minoxidil, or -PGE_2_ groups. Black circles indicate key decision points where the differentiation trajectory branches into multiple possible fates. Grey circles represent sequential progression of cells through differentiation, without any major fate change. The numbers label the sequential order of the cell states with state 1 the earliest, and higher numbers representing later stages. **(B–D and F–H)** The DEGs from the scRNA data were compared among various non-mesenchymal cells, including hematopoietic subclusters, TEC subclusters, and the one endothelial subcluster isolated from E13**–**13.5 Tbx^1+/+^ and Tbx1^neo2/neo2^ embryos following the indicated carrier, minoxidil, and PGE_2_ treatments administered to the pregnant mice. Heat maps revealed the relative expression of the key transcripts for the following populations: (B) hematopoietic subclusters, (C) TEC subclusters with transcripts involved in thymopoiesis, and (D) Sox family members in TECs. **(E)** The expression scaling used in B–D. **(F–H)** The single endothelial subset was compared for the following: (F) Sox family member TFs, (G) additional transcriptional regulators and, (H) angiogenic genes. The DEGs shown in B–D and F–H were selected based on a P < 0.00001, and logbase2 differences >1.0 were shown.

The mesenchymal subcluster representation of Sox family TFs suggested an amended developmental program in Tbx1^neo2/neo2^ embryos. To further assess this, a trajectory inference was done with Seurat’s Monocle3 integration software. The wild-type mesenchymal subsets (M1–M6) had a streamlined differentiation trajectory, with limited branch points (Fig. 5 A). Tbx1^neo2/neo2^ mesenchymal trajectories had extensive branching and differentiation paths, particularly with the M-5 subset. Minoxidil-treated mutants exhibited a less streamlined and shortened differentiation trajectory along with reduced branch points, indicating partial restoration of the mesenchymal differentiation program. Interestingly, PGE_2_ was more effective than minoxidil in re-equilibrating the trajectories (Fig. 5 A).

**Figure S4.**
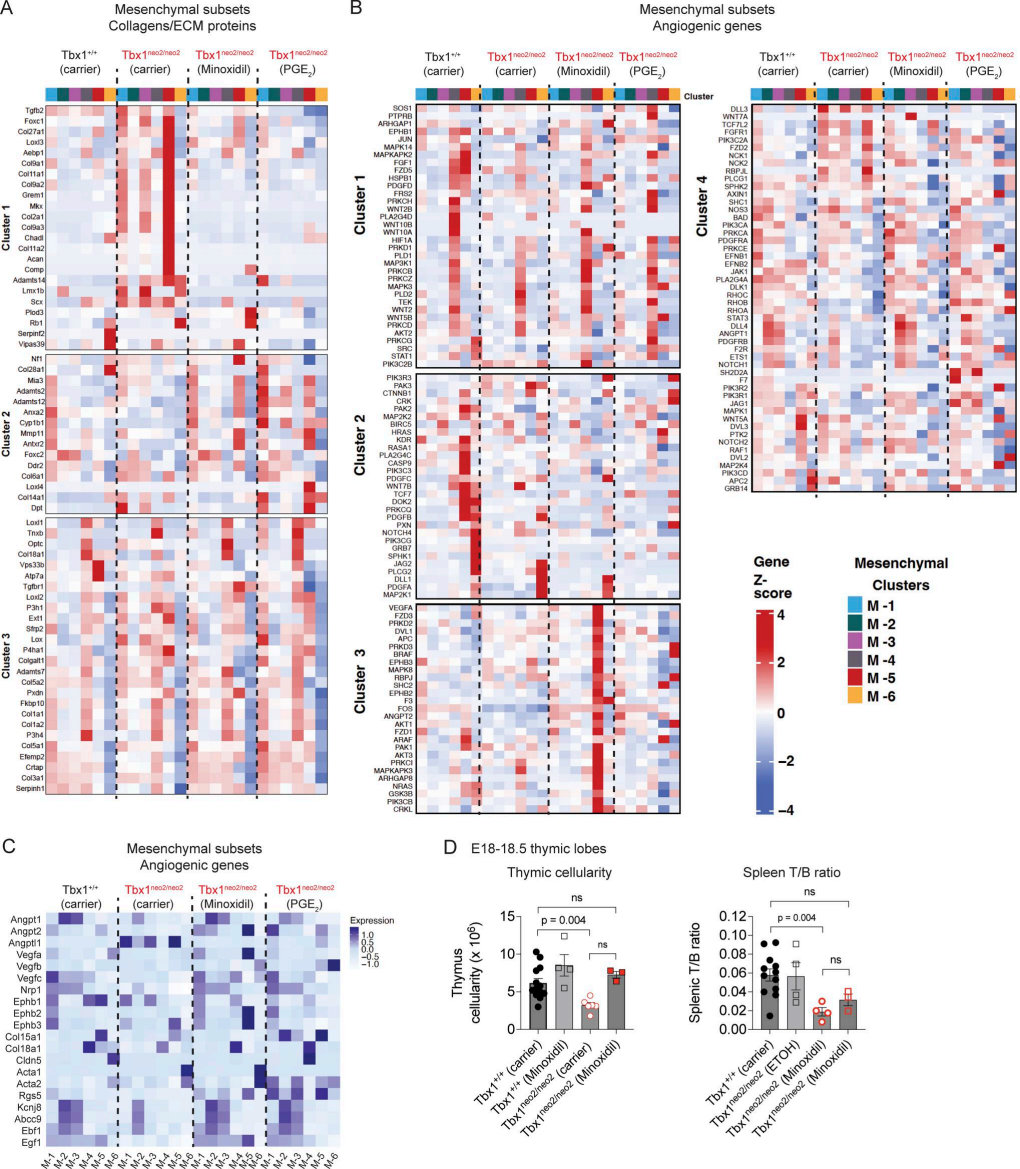
**Collagens, ECM, and angiogenic transcripts are altered in the six mesenchymal cell subsets from hypoplastic lobes. (A and B)** Fetal thymuses were isolated from Tbx1^+/+^ and Tbx1^neo2/neo2^ E13–13.5 embryos from carrier, minoxidil-, and PGE_2_-treated pregnant mice. ScRNA Seq revealed substantial transcriptome differences with the six mesenchymal cell clusters. **(A)** A heat map was used to visualize collagen and ECM transcript differences among the six mesenchymal clusters between control (Tbx1^+/+^-carrier) versus hypoplastic lobes from the (Tbx1^neo2/neo2^-carrier) and the corresponding genotypes following in vivo minoxidil or PGE_2_ administration. These were separated into three distinct clusters. **(B)** A separate heat map was established comparing the transcript levels for genes coupled to angiogenic pathways. These were separated into four clusters. The relative z-score and identification of the mesenchymal subclusters are illustrated. **(C)** Heat maps reveal the relative expression of the indicated mesenchymal transcripts grouped under angiogenic regulators. In A–C, genes with P < 0.00001 and logbase_2_ differences >1.5 are shown. **(D)** The thymic cellularity and splenic T/B cell ratios were determined from E18–18.5 embryos obtained from the indicated carrier and minoxidil treatment groups. Tbx1^+/+^-carrier (*n* = 15), Tbx1^+/+^-minoxidil (*n* = 4), Tbx1^neo2/neo2^-carrier (*n* = 6), and Tbx1^neo2/neo2^-minoxidil (*n* = 3). Statistical analyses was performed with one-way ANOVA (Brown–Forsythe and Welch tests).

Mesenchymal cells, TECs, and endothelial cells have significant interactions in the developing thymus (64). To examine the impact of the mesenchymal changes, the scRNA Seq data were analyzed to compare the hematopoietic, TEC, and endothelial subclusters. For most transcripts, the hematopoietic and TECs subsets were very similar among all groups assessed (Fig. 5, B and C). However, multiple Sox family TFs were upregulated in TEC and endothelial subclusters. Sox5, Sox6, Sox9, and Sox11 were higher in E-2, while Sox8, Sox11, and Sox13 were increased in E-4 in the hypoplastic lobes (Fig. 5 D). The E-5 population from the hypoplastic lobes also had increased Sox8, Sox11, and Sox13 (Fig. 5 D). Although Sox9 and Sox11 upregulate collagens and ECM transcripts in chondrocytes, the E-5 epithelial population did not have elevated collagen or ECM transcripts, suggesting the TEC cell type–specific gene signature remained unchanged. An additional adjustment that occurred selectively with minoxidil was a significant reduction in E-5 cells, preventing its annotation by the Seurat software (Fig. 5, C and D). The endothelial subset from the Tbx1^neo2/neo2^ embryonic thymus had increases in Sox7, Sox8, Sox9, and Sox11, while Sox10 was lower (Fig. 5, F–H). These findings suggest a common trend of Sox upregulation in multiple subsets. In TECs, minoxidil and PGE_2_ treatments normalized the Sox expression patterns. However, there were some differences between minoxidil and PGE_2_. Minoxidil reduced Sox9 and Sox11, but Sox5, Sox6, Sox13, Sox17, and Sox18 increased. PGE_2_ treatments reduced all the Sox family members, with some even lower than control embryos (Fig. 5 F). These findings demonstrate the Sox family of TFs undergo dynamic changes during thymic tissue formation. Additional TFs and other genes were altered in the endothelial population in the Tbx1^neo2/neo2^ group (Fig. 5, G and H). Tcf12, Runx2, and Hoxa7 were increased, while Fos and Jun were lower. Several angiogenic transcripts were reduced in expression, including angiopoietin (Angpt1 and Angpt2), vascular endothelial growth factor C (Vegfc), and ephrin receptors (Ephb1–3) (Fig. 5 H). An anti-angiogenic protein, Angptl1, was upregulated (Fig. 5 H). Angptl1 was also higher in 4 of 6 mesenchymal subsets (Fig. S4 C). Most of these angiogenic genes normalized following drug treatment (Fig. 5 H; and Fig. S4, B and C). Since many angiogenic genes were similar in wild-type and Tbx1^neo2/neo2^ embryos, a widespread vascular alteration of the thymus was not expected. In summary, thymic hypoplasia in the mouse model of 22q11.2DS causes a shift in mesenchymal trajectories, increasing those with chondrogenic potential.

### Disrupted thymic vasculature in embryonic mouse models of 22q11.2DS restored with minoxidil drug treatments

**Figure 6.**
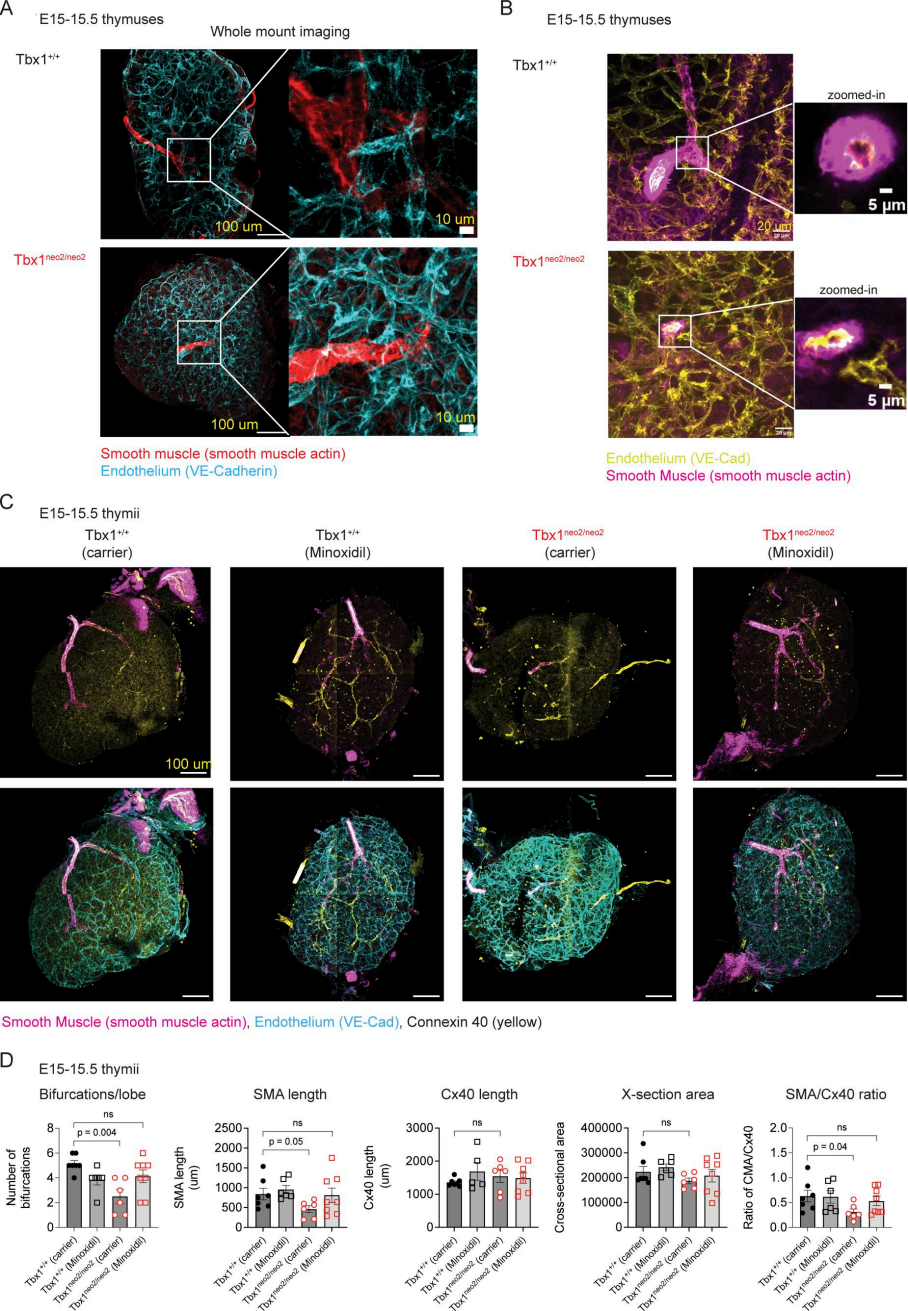
**Vascular changes evident in the hypoplastic thymic lobes improve following minoxidil administration. (A)** Intact thymic lobes from E15–15.5 embryos (Tbx1^+/+;+/neo2^ and Tbx1^neo2/neo2^) were stained with antibodies specific for the endothelium (VE-cadherin [VE-Cad]) and vascular smooth muscle (SMA). Whole-mount imaging and stitching was used to recreate the entire lobe, revealing arterial bifurcations. **(B)** The images were expanded to reveal a specific bifurcation with the levels of VE-Cad within and surrounding the arteriole shown. The zoomed-in area is shown by the line connecting the region. This was a single slice, enabling the selection of a cross-sectional view. **(C)** E15–15.5 embryonic thymuses from the indicated carrier or drug-treated pregnant mice were processed for whole-mount imaging. Antibodies specific for VE-Cad (endothelium), SMA, and connexin 40 were used to compare the vasculature and morphology of the tissues. The composite image was created by stitching individual subsections of the image together. **(D)** The number of bifurcations per thymic lobe, SMA length, cross-section area, and ratio of SMA length to Cx40 staining intensity determined for control (Tbx1^+/+^; *n* = 7), hypoplastic lobes (Tbx1^neo2/neo2^; *n* = 6), and minoxidil treatment groups for wild-type (*n* = 6) and mutant (*n* = 8) shown. Statistical comparisons were performed with two-way ANOVA.

The endothelial transcriptome from the E13.5 hypoplastic thymuses (Tbx1^neo2/neo2^) had higher TF levels (e.g., Sox family members) plus variations in multiple angiogenic genes (Fig. 5). Such changes could affect thymic vasculogenesis and vascular remodeling, potentially contributing to a restricted thymic size. To define the arterial and capillary vessel branching along with mural cell patterning in the thymus, whole tissue mounts were analyzed. E15–15.5 was selected for the imaging as the arterial branching and capillary endothelial vessel formations are sufficiently developed to visualize. The thymuses were stained with antibodies specific for endothelium (VE-cadherin) and vascular smooth muscle actin (SMA). SMA is produced by mesenchymal cell–derived mural cells and is recruited to endothelial cells to form the arteries and smaller arterial branches (Fig. 6 A). In Tbx1^+/+^ thymuses, SMA staining was continuous, indicating proper vascular smooth muscle cell (VSMC) coverage around the arteries. In contrast, Tbx1^neo2/neo2^ mutants had a discontinuous SMA staining. VE-cadherin was normal to higher in the hypoplastic thymuses (Fig. 6 A). These findings could reflect insufficient smooth muscle support. Zoomed-in panels further illustrate that in wild-type Tbx1^+/+^ thymuses, SMA and VE-cadherin colocalized properly around arteries are needed to sustain the vascular structure (Fig. 6 B). The enlarged image on the right represents single slices, enabling a cross-section view through the vasculature. In thymuses from the Tbx1^neo2/neo2^ mutants, SMA expression was drastically reduced. This suggested diminished VSMC coverage, which could result in fewer arterial bifurcations. To examine this possibility, whole-mount imaging was performed on the thymic lobes. The arterial bifurcations, vascular integrity, and smooth muscle cell investment was assessed by staining for SMA, VE-cadherin, and Cx40 (Fig. 6 C). In wild-type Tbx1^+/+^ thymic lobes, an average of five covered bifurcations per lobe was found (range 4–6) (Fig. 6, C and D). Tbx1^neo2/neo2^ thymuses had only two SMA^+^ bifurcations per lobe (range 0–3). These improved to an average of four following minoxidil administration (Fig. 6, C and D). Total smooth muscle coverage of these arterial branches, measured by SMA length, was likewise significantly reduced in the Tbx1^neo2/neo2^ hypoplastic thymuses (Fig. 6 C). This improved following minoxidil treatments. Arterial endothelial vessels, identified by dual positive staining of connexin 40/VE-cadherin, appeared similar in the wild-type (Tbx1^+/+^) and hypoplastic (Tbx1^neo2/neo2^) lobes (Fig. 6, C and D). However, there were significant reductions in smooth muscle cell investment around the arteries in hypoplastic lobes, as measured by the ratio of SMA+ length/connexin 40 length. These findings extend the transcriptomic data with E13–13.5 thymuses, providing functional evidence of vascular changes at E15–15.5. Vascular changes in the Tbx1^neo2/neo2^ thymuses were linked to mesenchymal alterations (chondrogenic patterns) with dysregulated (ECM) remodeling, collagen increases, and angiogenic factor changes.

### Pth malformations corrected by minoxidil and PGE_2_ treatments

**Figure 7.**
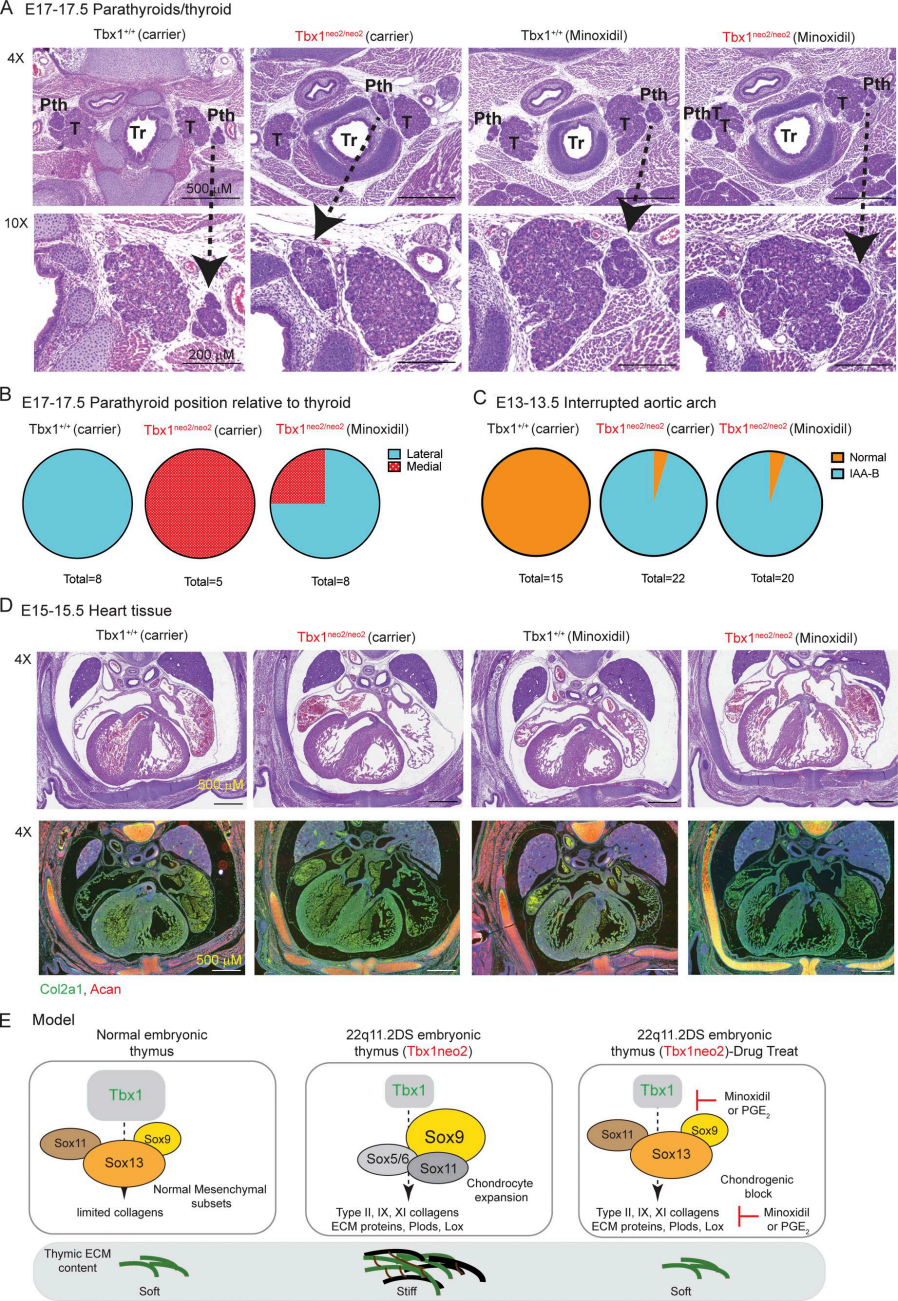
**The abnormal medial positioning of the Pth’s in Tbx1** ^**neo2/neo2**^ **embryos corrected following *in utero* minoxidil administration. (A)** Timed pregnant mice from Tbx1^+/neo2^ intercrosses received carrier or minoxidil treatments as in Fig. 2 A. At E17–17.5, embryos from the indicated genotypes were sectioned and stained with H&E to visualize the trachea (Tr), thyroid (T), and Pth locations. The positions of the different organs are shown. Both 4× and 10× images were taken for the trachea (Tr), thyroids (Th), and Pth. Arrows are shown for the Pth structures in the 4× vs. 10× images. Data are representative of 8, 5, and 8 embryos from Tbx1^+/+^ (carrier), Tbx1^neo2/neo2^ (carrier), and Tbx1^neo2/neo2^ (minoxidil) genotypes, respectively. **(B)** Pie charts reveal the lateral (blue) and medial (red) distributions of the Pth’s in the three groups of embryos. **(C)** Pie charts reveal the number of E13–13.5 embryos with a normal aortic arch (orange) or those with a visually detectable IAA-B (blue). This was shown for Tbx1^+/+^, Tbx1^neo/neo2^, and Tbx1^neo2/neo2^ genotypes with carrier or minoxidil treatments. Tbx1^+/+^-carrier (*n* = 15), Tbx1^neo2/neo2^-carrier (*n* = 22), and Tbx1^neo2/neo2^-minoxidil (*n* = 20). **(D)** Heart sections were prepared from the indicated E15–15.5 cohorts of embryos, obtained from pregnant mice with/without minoxidil administration, and stained with H&E. Sections were also processed for IHC to detect Col2a1 and Acan protein expression. The images are representation of >6 embryos/group. **(E)** Model depicting the formation of the thymus in normal and 22q11.2 settings. Minoxidil administration reduces the expression of chondrogenic collagens (Col2a1, Col9a1, and Col11a1) and ECM cross-linking enzymes (Plod and Lox), decreasing ECM stiffness. This reduces the chondrogenic potential of mesenchymal cells, enabling restoration of thymus growth.

The thymus and Pth’s coordinately develop within the third pharyngeal pouches during embryogenesis and then separate at E12 (65). Their patterning depends on surrounding NCC-derived mesenchymal cells producing Sonic hedgehog and bone morphogenic protein 4 that delineate the dorsally and ventrally located Pth and thymus, respectively (47). In a previous report, Tbx1 heterozygous (Tbx1^+/−^) E18.5 embryos had smaller-sized Pth’s that were displaced and localized between the trachea and thyroid (66). In our analysis of E17–17.5 Tbx1^neo2/neo2^ embryos using transverse sections, the Pth was also incorrectly positioned, medial to the thyroid, unlike the control embryos where the Pth is laterally positioned (Fig. 7, A and B). To determine if minoxidil could correct this positioning, pregnant mice were administered this drug at E8, E9, and E10, and the embryos were isolated at E17–17.5 (Fig. 2 A). The Pth was correctly repositioned lateral to the thyroid in six of eight8 embryos (Fig. 7, A and B). One report indicated that the septation defects of the heart improved following minoxidil treatments (67, *Preprint*). Consequently, we assessed if the penetrance of the interrupted aortic arch type B (IAA-B), a common heart defect in 22q11.2DS patients, was altered after minoxidil treatments. IAA-B was 90% penetrant in the Tbx1^neo2/neo2^ embryos regardless of whether minoxidil or PGE_2_ were administered (Fig. 7 C). We then used histology to monitor correction of the septation of the heart following minoxidil administration. No septation defects were observed in the heart, which appeared like wild type (Fig. 7 D). Moreover, minoxidil had no effect on the overall structure of the heart, with similar staining patterns of Col2a1 and Acan (Fig. 7 D). In summary, our findings indicate that the elevated production of collagens and ECM proteins contribute to congenital malformations linked to the third pharyngeal pouch in 22q11.2DS mouse models, which are correctable by minoxidil administration.

## Discussion

**Figure S5.**
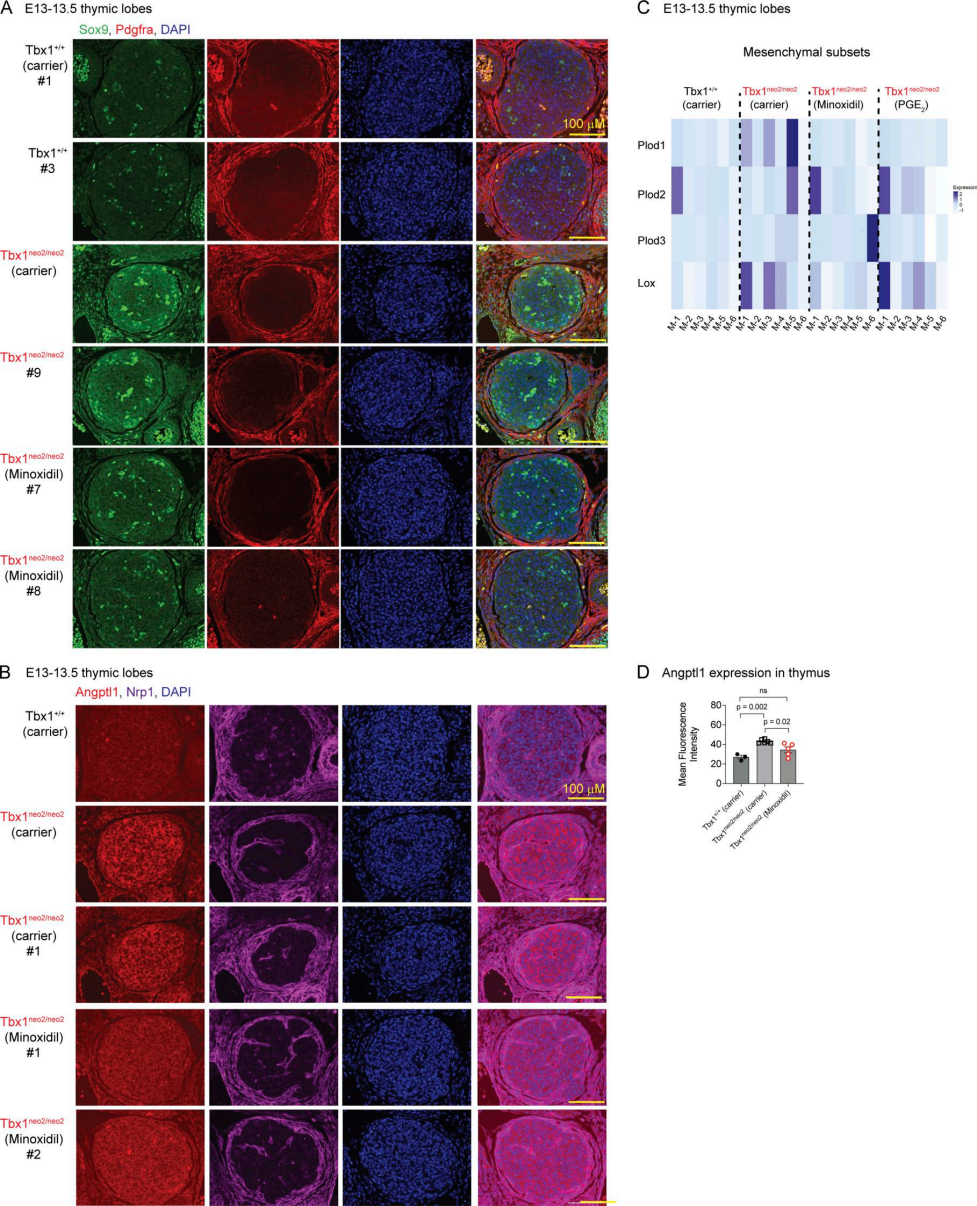
**Elevated levels of Sox9 and Angptl1 levels along with Plods and Lox are normalized in Tbx1** ^**neo2/neo2**^ **thymic lobes following minoxidil treatments. (A)** E13.5 thymuses were processed for immunofluorescence using wild-type (Tbx1^+/+^, *n* = 2), Tbx1^neo2/neo2^-carrier (*n* = 4), and Tbx1^neo2/neo2^-minoxidil treatment groups. The drug treatment is listed under the genotype name, and the embryo identifier is shown with a # sign. Staining was done with antibodies detecting Sox9 (green), Pdgfra (red), and DAPI (purple), the latter used to detect the nucleus. Confocal microscopy was performed to more precisely delineate Sox-9 expression in the indicated embryonic thymuses. Some of the images are the same as those in Fig. 4 B, with individual staining shown in this Supplemental figure. The composite was provided in Fig. 4 B. **(B)** The indicated embryos were done with antibodies specific for Angptl1 (red) and Nrp1 (purple), along with a nuclear marker (DAPI, blue), to compare the levels of Angptl1. Immunofluorescence was used to visualize these levels. Sections are representative of five independent samples/genotype. **(C)** A heat map was established comparing the transcript levels in mesenchymal cells for genes coupled to Plods and lox, enzymes involved in collagen cross-linking. These were separated into four clusters. The relative z-score and identification of the mesenchymal subclusters are illustrated. Genes with P < 0.00001 and logbase_2_ differences >1.5 are shown. **(D)** Quantitation of Angptl1 levels was performed by comparing the mean fluorescent intensity of a defined area among all the groups analyzed. Statistical analyses were done using one-way ANOVA. This was determined with thymuses from Tbx1^+/+^-carrier (*n* = 3), Tbx1^neo2/neo2^-carrier (*n* = 6), and Tbx1^neo2/neo2^-minoxidil (*n* = 5) groups.

A dysregulation of NCC-derived progenitor mesenchymal cells in 22q11.2DS was hypothesized to be causal to congenital malformations affecting the pharyngeal pouches and arteries four decades ago (24, 68). Our current manuscript confirms this hypothesis and reveals the mechanism by which NCC-derived mesenchymal changes affect thymus expansion in 22q11.2DS (24, 32, 33). In the developing thymus, mesenchymal subsets form the capsule and trabeculae, ultimately becoming postnatal fibroblast subtypes (64, 69). The mesenchymal cells also differentiate into mural cells, which form the VSMCs and perivascular cells surrounding the endothelial vessels. Between E12–E13.5, there are six mesenchymal subclusters present in both normal and hypoplastic thymic lobes (Tbx1^neo2/neo2^ embryos) (Fig. 2). The small thymuses from embryonic mouse models of 22q11.2DS (Tbx1^neo2/neo2^) had an overrepresentation of NCC-derived chondrocyte-like cells (M-5) and mesoderm-derived mesenchymal cell signatures (M-6). In the M-5 population, the trio of Sox5, Sox6, and Sox9 family members and Sox11 were elevated. This cadre of TFs controls the differentiation and programming of mesenchymal subsets into chondrocytes (70, 71). Other Sox TFs differentially impacted were Sox 8, 9, upregulated in one or more of the thymic mesenchymal cell subsets (M-5), and Sox10, which was diminished (Fig. 3). Sox9 transcriptionally activates type II collagens (Col2a1) that form the fibers. This TF also drives type IX (Col9a) and XI (Col11a) collagen expression, increasing the tensile strength of the fibers, along with aggrecan (Acan) that cross-links the fibers (72, 73). All these transcripts were higher in the Tbx1^neo2/neo2^ embryonic thymuses (Figs. 1 and 3). Immunofluorescence confirmed elevated levels of Sox9 along with Col2a1 and Angptl1 in hypoplastic embryonic thymuses and from postnatal human 22q11.2DS thymuses (Figs. 4 and S5) (32). In the setting of a hypoplastic thymus, increased levels of these specific collagens and aggrecan are predicted to create a more rigid and less elastic tissue, which could explain the size restriction. This was also evident in the thicker capsule. Sox9 also diminishes VSMC contractility (74). The reduced vascular smooth muscle accumulation in the hypoplastic Tbx1^neo2/neo2^ lobes was consistent with higher Sox9 levels and the downregulation of SMA (Act2) along with Myh11 and Cnn1 (Fig. S4). This would result in fewer neural crest–derived mesenchymal cells adopting a smooth muscle fate. In addition, the higher levels of Sox11 (SoxC family member) would also increase ECM protein deposition. Sox11 participates in both neurogenesis and skeletogenesis (75). Furthermore, Sox11 may affect thymic tissue remodeling, as it is involved in epithelial–mesenchymal interactions (61). In summary, the Sox TF changes would impact several mesenchymal subsets. Interestingly, all the mesenchymal subsets from the hypoplastic lobes had altered transcripts. This suggests that the NCC-derived mesenchymal cells have an amended developmental program in 22q11.2DS prior to the establishment of the thymic anlage. This concurred with the trajectory inference analyses. Thus, Tbx1^neo2/neo2^ mesenchymal trajectories had more extensive branching and differentiation paths than the controls. Minoxidil-treated mutants exhibited a less streamlined and shortened differentiation trajectory along with reduced branch points, indicating partial restoration of the mesenchymal differentiation program. Interestingly, PGE_2_ was more effective than minoxidil in re-equilibrating the trajectories (Fig. 5 A). Based on previous reports, one plausible driver of the mesenchymal changes in 22q11.2DS is an upregulation of retinoic acid (RA) receptor pathways (21, 76, 77). We found that multiple RA targets (Sox5, Sox6, Sox9, Sox11, Runx2, and Hoxa7) were upregulated in E13 Tbx1^neo2/neo2^ hypoplastic thymuses and control mesenchymal cell differentiation, migration, and chondrogenic expansion (21, 36, 37, 38, 78, 79, 80, 81, 82). In addition to the mesenchymal subset changes, the endothelial population had elevated Sox family members, collagens, and increased levels of Angptl1 in the hypoplastic thymuses. Angptl1 antagonizes angiogenesis, consistent with a reduced number of arterial bifurcations in the hypoplastic thymuses (Fig. 6) (83). Whole-mount imaging confirmed that the hypoplastic thymuses were spatially restricted. Other studies have shown that the endothelial cell barrier, formed using induced pluripotent cells from 22q11.2DS patients, have increased leakiness and vascular permeability compared with non-22q individuals (84, 85, *Preprint*).

The transcriptional alterations in hypoplastic thymuses from the 22q11.2 mouse models were normalized following minoxidil and PGE_2_ treatments, done in pregnant mice prior to thymic tissue specification. In particular, Sox family members and their corresponding targets, collagens and lysyl- hydroxylases, were restored to more normal values. Minoxidil treatment eliminated the chondrocyte overrepresentation. These experiments further imply that the drug influences mesenchymal cell fate prior to formation of the thymic anlage. This was confirmed by both the transcriptome and immunofluorescence data. Minoxidil was first developed as a vasodilator to treat patients with hypertension, relaxing vascular smooth muscle by blocking ATP-sensitive potassium channels (86). While this may have contributed to an improved thymic vasculature for the Tbx1^neo2/neo2^ embryos, our data argue the improved expansion of the thymus in the Tbx1^neo2/neo2^ embryo results primarily from reduced collagen and ECM protein accumulation. Minoxidil treatment reduced the expression of chondrogenic collagens (Col2a1, Col9a1, and Col11a1) and ECM cross-linking enzymes (Plod and Lox), which was evident in the diminished thickness of the thymic capsule (Fig. S5 C). The softening of the ECM due to minoxidil prevents a feedforward signal that would enhance Sox9 expression, thereby reducing the expansion of chondrogenic-fated cells (87). Consequently, mesenchymal differentiation is reprogrammed away from the pathological chondrogenic state, restoring a transcriptional environment more supportive of thymopoiesis and normal T cell output (Fig. 7 E). Our interpretation was further supported with data that vasodilators, blood pressure reduction, or adrenergic receptor antagonists had no effect on the size of the hypoplastic embryonic Tbx1^neo2/neo2^ thymuses (Table 1).

Despite the noted size restriction of the hypoplastic thymuses in 22q11.2DS, the progression of thymocytes from the DN to DP and then SP subsets appeared normal. This agrees with TEC transcriptome data, wherein the key transcripts involved in thymopoiesis were expressed at similar levels in control and hypoplastic thymuses (Fig. 5 C) (32). One unexpected finding in the TEC populations was elevated levels of different Sox family members in the E-2 and E-5 subsets of TECs (Fig. 5 D). E-2 subset had a number of “mesenchymal” transcripts, which could suggest direct mesenchymal to epithelial transitions. The two bipotent progenitor populations of TECs in normal thymuses do not have these mesenchymal markers (88). This would indicate that the development programming in TECs from 22q11.2DS could be modulated by the Sox TF changes. This might even impact the establishment of the medullary TEC mimetics that are required for T cell tolerance to tissue-specific proteins (89). Consistent with this, 22q11.2DS patients have a higher incidence of autoimmunity relative to the non-22q groups (5, 6). Yet, most studies have reported that T cell development is normal in patients with 22q11.2DS and in various distinct mouse models of 22q11.2DS (6, 27, 32, 49, 90). While one study suggested that there were differences in T regulatory cell suppressor activities, subsequent results indicated the T regulatory cells were normal (23, 90). It is unclear whether the thymic hypoplasia is solely responsible for the peripheral T cell lymphopenia. For example, we noted that minoxidil treatments in pregnant mice (E8–E10) normalized the size of the E18–18.5 thymus in the Tbx1^neo2/neo2^ cohort like controls (Fig. S4 D). Yet, there was only a slight improvement in the splenic T to B cell ratio when comparing carrier versus drug-treated e18–e18.5 Tbx1^neo2/neo2^ embryos (Fig. S4 D). Current experiments are addressing additional causes for the peripheral T cell lymphopenia seen in patients.

22q11.2DS impacts the thymus, Pth’s, and aortic arch. The administration of minoxidil in utero corrected the improper medial positioning of the Pth’s relative to the thyroids in the Tbx1^neo2/neo2^ embryos (Fig. 7). This is the first evidence that mesenchymal cell defects are directly consequential to the Pth changes in 22q11.2DS. While septation defects in the hearts of Tbx1^+/LacZ^ embryos were reported (distinct 22q mouse model, 50% wild-type Tbx1 expression), the Tbx1^neo2/neo2^ line did not appear to have such problems (67, *Preprint*). Moreover, our experiments also indicated that the doses and time of administration of minoxidil or PGE_2_ in utero did not correct the IAA-B, the most common cardiac phenotype in 22q11.2DS patients (9). Either earlier administration of higher doses and/or more prolonged drugs may be needed to correct the various congenital heart defects.

The congenital defects due to 22q11.2 deletion overlap substantially with those from coloboma, heart defects, atresia choanae, growth retardation, genital abnormalities, and ear abnormalities and otofaciocervical syndromes, gestational diabetes, and RA embryopathies (3, 28, 47, 91, 92). These shared congenital problems are linked to developmental issues within the pharyngeal region. We propose these are connected by transcriptional changes within the mesenchymal cell subsets. This notion is supported by evidence in pregestational diabetic mouse models, wherein diabetes impacts selected mesenchymal subsets, leading to the congenital heart defects (93). Increased RA is a common link among several of these syndromes (76, 77, 94, 95). In summary, mesenchymal cell dysfunction contributes to several congenital malformations arising from 22q11.2DS that impact the thymus, Pth’s, and velopharyngeal regions. Several drugs were identified that reduced the severity of these congenital problems, providing a possible clinical approach for 22q11.2DS patients.

## Materials and methods

### Study approval

Animal work described in this manuscript has been approved and conducted under the oversight of the UT Southwestern Institutional Animal Care and Use Committee *(*APN numbers 2015-101163 and 2015-101247). For 22q11.2DS and control patient samples, the Institutional Review Board at UT Southwestern Medical Center approved this study (# 072010-009; IRB, # 112010-013; IRB). Consent was obtained for collecting thymic tissues from cardiac patients, some of whom were subsequently determined to carry 22q11.2DS.

### FTOCs and RTOCs

FTOC assays were performed as previously described (32, 96). The media consisted of RPMI supplemented with 20% fetal calf serum and HEPES, L-glutamine, sodium pyruvate, penicillin, streptomycin, 5 × 10^−5^ M 2-mercaptoethanol, and nonessential amino acids. RTOCs were done with normal and hypoplastic fetal thymic lobes, isolated at gestational ages E13.0–13.5. A minimum of four hypoplastic lobes were needed for a single RTOC assay (>15,000 cells total). Lobes were washed with PBS and digested in 0.25% trypsin and 0.02% EDTA for 4–10 min at 37°C; the stopping point determined as a single-cell suspension became evident. Trypsinization was terminated by washing the cells in serum-containing media. The single-cell suspensions were stained with antibodies specific for mesenchymal cells (Pdgfra-PE) and TECs (EpCAM-FITC), done under sterile conditions. After 20-min incubations, the cells were washed and sorted into three populations: mesenchymal, epithelial, and the remaining cells (EpCAM^−^Pdgfra^−^) that contained ETPs, dendritic cells, endothelial cells, and macrophages. RTOC assays were performed by reaggregating the three cell groups: EpCAM^+^ cell (∼30%), Pdgfra^+^ (∼30%), and EpCAM^−^ Pdgfra^−^ (∼40%) in a 1.5-ml tube. Cells were centrifuged consecutively for 5 and 10 min at 1,000 and 2,000 rpm, respectively. After the second spin, the supernatant was removed, leaving behind 2–5 μl of aggregated cells, which was placed on ice for 10 min. The cell pellet was gently dispersed and drawn into a pulled glass pipette. A single drop of aggregated cells was applied onto a Millipore nitrocellulose filter, the latter on a sterile foam sponge (2-mm thick) in a single well of a 6-well tissue culture plate. The cells were cultured and analyzed as described (32, 97). In certain experiments, the cultured media was supplemented with various concentrations of either minoxidil (3–10 μM), PGE_2_ (100–1,000 nM), labetalol (300 nM), enalaprilat (100 μM), nifedipine (100–300 nM), or hydralazine (50–100 nM). Fresh media containing either the carrier (5% ETOH) or the drugs were replenished every 2 days.

### In vivo drug treatments

On the day of mouse intercrosses, Tbx1^+/neo2^ male mice were co-housed with Tbx1^+t/neo2^ female mice after 4 p.m. The next morning, females with evidence of a vaginal plug were designated as day e0–0.5 post coitus. We provide this range throughout the manuscript, as a 0.5-day difference can significantly affect the thymic cellularity when comparing hypoplastic and normal lobes and affect DP numbers when assessed at E17–17.5. Timed pregnant mice were injected intraperitoneally with either an ETOH carrier (5% ETOH in PBS), minoxidil (200 μl, 0.55 mg/ml), PGE_2_ (90 μl, 3.6 ng/μl), or enalapril maleate (100 μl, 1 μg/ml) in the mornings at E8, E9, and E10. Embryos were isolated from the pregnant mice at E13–13.5, E15–15.5, or E17–17.5 post coitus.

### Tissue staining

E13.5–E15.5 whole embryos (for thymus and heart) and E17–17.5 (jaw to upper abdomen cut) embryos (for Pth) were fixed for 24 h in 4% paraformaldehyde (in PBS) at 4°C. The tissues were dehydrated in a stepwise ethanol gradient of 25, 50, 75, and 100% ethanol, prepared in PBS when diluted. After a subsequent wash in xylene, the tissues were embedded in paraffin and sectioned (10-μm thick). Slides were de-paraffinized in xylene and rehydrated using a descending ethanol gradient (100, 95, 90, 80, 70, and 50% ethanol). Antigen retrieval was performed for 15 min at 95°C in Antigen Retrieval R Buffer A pH 6 (Electron Microscopy). Slides were blocked in CAS Block (Invitrogen) for 2 h at room temperature (RT). The antibodies used for staining are listed in Table S3 and the figure legends. In most experiments, the antibodies were incubated with the slides overnight (O/N) at 4°C. Secondary antibodies (Invitrogen) were used as recommended by the manufacturer. Certain slides were stained with DAPI to delineate the nucleus (Molecular Probes) prior to mounting with Prolong Gold anti-fade Reagent (Invitrogen). Images were taken on a Laser scanning confocal Zeiss LSM880 inv. + 2-Photon and Keyence Fluorescence microscope. Some images were taken on a Leica TCS SP5 confocal microscope. Images were analyzed using updated ImageJ software termed Fuji.

For the whole-mount staining, E15.5 whole thymuses were fixed with 4% PFA for 1 h at RT. Fixed thymuses were permeabilized in 1% Triton-X in PBS for 1.5 h and then blocked in CAS block (Invitrogen) for 1.5–2 h. Thymuses were then incubated with indicated antibodies dissolved in CAS block O/N at 4°C. Thereafter, thymuses were washed thrice in PBS and then incubated with appropriate secondary antibody (1:250; Invitrogen) dissolved in CAS block O/N at 4°C. Antibody information can be found in Table S2. The next day, the thymuses were washed in PBS, sequentially dehydrated into 100% methanol, and visualized after clearing in 2:1 benzyl alcohol: benzyl benzoate (BABB). Thymuses were mounted in concavity slides in BABB. Images were obtained using Zeiss LSM700 Axio Imager confocal microscope. The SMA stain was manually traced in Fuji software (ImageJ), and the number of bifurcations was counted. All SMA+ vessels inside the thymus were added together to measure SMA+ arterial length.

### scRNA Seq information and data analysis

Cell Ranger’s (v5.0.1, 10X Genomics) mkfastq module was used to convert BCL files to FASTQ format. Reads from FASTQ files for each library were aligned to the mouse reference genome (mm10), and the transcript counts of each cell were quantified using unique molecular identifier (UMI) and valid cell barcode. The gene expression matrix generated from cell ranger count module was then used as input to Seurat R package (v4.0.1) for the downstream analysis (98). Cells with <200 genes per cell and very high mitochondrial gene content were filtered out. Global-scaling normalization method defined as “Log Normalize” was used. Subsets of genes exhibiting high variation across the single cells were compared. The highly variable genes were calculated using the “FindVariableFeatures” module in Seurat. In this module, average expression and dispersion per gene were calculated, and features were divided into bins to get z-scores for dispersion per bin. “FindIntegrationAnchors” and “IntegrateData module” in Seurat were then used to find anchors and integrate Seurat objects corresponding to sample names Tbx1^+/+^ (carrier), Tbx1^neo2/neo2^ (carrier), Tbx1^neo2/neo2^ (minoxidil), and Tbx1^neo2/neo2^ (PGE_2_). Seurat integrated analysis was performed across all samples. Data were then scaled, and dimensional reduction was performed with principal component analysis. For the sample, a shared nearest neighbor (SNN) graph was constructed with the “FindNeighbors” module in Seurat by determining the k-nearest neighbors of each cell. The clusters were then identified by optimizing SNN modularity using the “FindClusters” module. This allowed for a sensitive detection of rare cell types. We obtained 19 clusters with a resolution of 0.3. Uniform manifold approximation and projection (UMAP) plots were generated using the DimPlot module in Seurat. Each cluster was compared with all other clusters using Wilcoxon rank-sum test to test for significant differentially expressed genes. The genes identified as relatively overexpressed in a cluster as compared with all other cells were termed as “markers”. Clusters were named based on gene markers specific to various cell types. Differential gene expression testing was performed using “FindMarkers” module in Seurat between normal fetal thymus (Tbx1^+/+^) and 22q.11.2DS hypoplastic lobes (Tbx1^neo2/neo2^) minoxidil, PGE_2_, or ETOH as a carrier control. Dot plot was generated using DotPlot function in Seurat. Heat maps were generated using the “DoHeatmap” function in Seurat. The scRNA Seq dataset has been deposited in the Gene Expression Omnibus database (GEO accession #GSE272825). Monocle3 (https://cole-trapnell-lab.github.io/monocle3/docs/trajectories/) was used to study the trajectory of mesenchymal cells. Monocle tracks the magnitude of transcriptional change that a cell undergoes starting from root to the end state defined as pseudotime. In this analysis, M-1/M-3 was defined as the root in trajectory analysis.

### Online supplemental material

The supplementary information provides additional insights into the cause of thymic hypoplasia in 22q11.2DS mouse models. Five figures, three tables, and a supplementary spreadsheet containing the gene expression changes are included as follows: Fig. S1 shows the human 22q11.2 locus and corresponding mouse models that have overlapping congenital features along with RTOC procedures. Fig. S2 shows the differential effects of PGE_2_ versus minoxidil in FTOCs and RTOCs. Fig. S3 shows that the minoxidil and PGE_2_ have beneficial growth effects in vivo on developing thymuses. Fig. S4 shows that the collagens, ECM, and angiogenic transcripts are altered in the six mesenchymal cell subsets from hypoplastic lobes. Fig. S5 shows the elevated levels of Sox9 and Angptl1 levels along with Plods and Lox are normalized in Tbx1^neo2/neo2^ thymic lobes following minoxidil treatments. Table S1 shows the mouse models of 22q11.2DS. Table S2 shows the scRNA Seq data summary. Table S3 shows the reagents and supplies used in the study. Data S1 shows the gene expression changes in E13–13.5 thymic cell subsets resulting from minoxidil.

## Supplementary Material

Table S1 shows the mouse models of 22q11.2DS.

Table S2 shows the scRNA Seq data summary.

Table S3 shows the reagents and supplies used in the study.

Data S1 shows the gene expression changes in E13–13.5 thymic cell subsets resulting from minoxidil.

## Data Availability

The data underlying Figs. 1, 2, 6, and 7 are available in the published article and in the online supplemental material. The data underlying Figs. 3 and 5 are openly available in the scRNA Seq dataset deposited in the GEO database (GEO accession #GSE272825).
