# Supplementary material for: Minoxidil restores thymic growth in 22q11.2 deletion syndrome by limiting Sox9+ chondrocyte expansion
Source: J Hum Immun. 2025 Aug 12;1(3):e20250143. doi: 10.70962/jhi.20250143 (PMC12829771; doi:10.70962/jhi.20250143)
Supplement: Table S1 — shows the mouse models of 22q11.2DS. [file jhi_20250143_tables1.docx]

Supplementary Table 1. Mouse models of 22q11.2DS

| **Supplemental Table 1**: Mouse models of 22q11.2DS | | | | |
| --- | --- | --- | --- | --- |
| Mouse Line | Relative Tbx1 levels^a^ | Thymus | Neonatal Lethality | Heart defects |
| Tbx1^+/+^ | 1.0 | Normal | No | No |
| Tbx1^+/neo2 b^ | 0.67 | Mild Hypoplasia | Low | Yes |
| Tbx1^+/-^ | 0.5 | Mild Hypoplasia | Low | Yes |
| Tbx1^+/neo^ | 0.5 | Mild Hypoplasia | Low | Yes |
| Tbx1^neo2/neo2^ | 0.35 | Severe hypoplasia | 100% | Yes |
| Tbx1^neo2/neo^ | 0.2 | Aplasia | 100% | Yes |
| Tbx1^-/-^ | 0 | Aplasia | 100% | Yes |
| Df(16)1/+; 1.5 Mb deletion | 0.5 | Mild Hypoplasia | Low | Yes |
| Del(3.0Mb)/+; 3 Mb deletion^b^ | 0.5 | Mild Hypoplasia | Low | Yes |

^a^Obtained from published reports

^b^See Supplemental Figure 1 for human chr 22q and murine chr 16
