# Supplementary material for: Minoxidil restores thymic growth in 22q11.2 deletion syndrome by limiting Sox9+ chondrocyte expansion
Source: J Hum Immun. 2025 Aug 12;1(3):e20250143. doi: 10.70962/jhi.20250143 (PMC12829771; doi:10.70962/jhi.20250143)
Supplement: Table S2 — shows the scRNA Seq data summary. [file jhi_20250143_tables2.docx]

Supplementary Table 2. Single cell RNA sequencing data summary

| **Supplemental Table 2**. Single cell RNA sequencing data summary | | | |  |
| --- | --- | --- | --- | --- |
| Embryonic thymus genotype | **Tbx1^+/+^ (carrier)** | **Tbx1^neo2/neo2^ (carrier)^a^** | **Tbx1^neo2/neo2^ (minoxidil)** | **Tbx1^neo2/neo2^ (PGE_2_)** |
| Estimated total # cells | 11,908 | 11,364 | 3362 | 4471 |
| Mean reads/cell | 61,572 | 50,544 | 258,813 | 150,320 |
| Median genes/cell | 4,515 | 3,584 | 6,017 | 5367 |
| Total # reads | 733,194,190 | 574,386,022 | 870,128,648 | 672,081,524 |
| Total genes detected | 21,946 | 21,648 | 21,410 | 21,269 |

^a^Hypoplastic thymic lobes
