# Supplementary material for: Minoxidil restores thymic growth in 22q11.2 deletion syndrome by limiting Sox9+ chondrocyte expansion
Source: J Hum Immun. 2025 Aug 12;1(3):e20250143. doi: 10.70962/jhi.20250143 (PMC12829771; doi:10.70962/jhi.20250143)
Supplement: Table S3 — shows the reagents and supplies used in the study. [file jhi_20250143_tables3.docx]

Supplementary Table 3. Reagents and supplies used in the study

| **Supplemental Table 3**. Reagents and supplies used in the study | | |
| --- | --- | --- |
| Reagent or Resource | Source | Identifier |
| Antibodies | | |
| Pdgfra (Goat = Gt) | R and D Systems | Cat# AF1062 RRID:AB_2236897 |
| Connexin-40(Rabbit =Rb) | Invitrogen | Cat#36-5000 RRID:AB_2533264 |
| VeCADH (Gt) | R and D Systems | Cat#AF1002, RRID:AB_2077789 |
| Smooth Muscle Actin (Mouse = Ms) | R and D Systems | Cat# MAB1420, RRID:AB_262054 |
| Sox9 (Rb) | Miilipore Sigma | Cat#AB5535 RRID:AB_2239761 |
| Col2a1 (Rb) | Proteintech | Cat#28459-1-AP RRID:AB_2881147 |
| Angptl1 (Ms) | Santa Cruz | Cat#sc-271841 |
| Nrp1 (Gt) | R and D Systems | Cat#AF566 RRID:AB_355445 |
| Donkey Anti-Rabbit IgG H&L (Alexa Fluor® 488) | Abcam | Cat# ab150073 RRID:AB_2636877 |
| [Donkey Anti-Rabbit IgG H&L (Alexa Fluor® 594)](https://www.abcam.com/donkey-rabbit-igg-hl-alexa-fluor-594-ab150076.html) | Abcam | Cat# ab150076, RRID:AB_2782993 |
| [Donkey Anti-Mouse IgG H&L (Alexa Fluor® 488)](https://www.abcam.com/donkey-mouse-igg-hl-alexa-fluor-488-ab150105.html) | Abcam | Cat# ab150105, RRID:AB_2732856 |
| [Donkey Anti-Mouse IgG H&L (Alexa Fluor® 594)](https://www.abcam.com/donkey-mouse-igg-hl-alexa-fluor-594-ab150108.html) | Abcam | Cat# ab150108, RRID:AB_2732073 |
| [Donkey Anti-Goat IgG H&L (Alexa Fluor® 594)](https://www.abcam.com/donkey-goat-igg-hl-alexa-fluor-594-ab150132.html) | Abcam | Cat# ab150132, RRID:AB_2810222 |
| [Donkey Anti-Rabbit IgG H&L (Alexa Fluor® 647)](https://www.abcam.com/donkey-rabbit-igg-hl-alexa-fluor-647-ab150075.html) | Abcam | Cat# ab150075, RRID:AB_2752244 |
| Anti-CD8-FITC | Tonbo Biosciences | Cat# 35-0081, RRID:AB_2621671 |
| Anti-CD4-PE | Thermo Fisher Scientific | Cat# 50-0041-80,RRID:AB_10596360 |
| Anti-TCR-b-PerCP-Cy5.5 | Tonbo Biosciences | Cat# 65-5961, RRID:AB_2621911 |
| Anti-CD69-APC | Thermo Fisher Scientific | Cat# 17-0691-82, RRID:AB_1210795 |
| Anti-CD45-PerCPCy5.5 | BioLegend | 103132 Clone 30-F11 |
| Anti-B220-APC | Tonbo Biosciences | Cat# 20-0452, RRID:AB_2621574 |
| Anti-CD44-APC | BD bioscience | Cat# 559250, RRID:AB_398661 |
| Anti-CD25-FITC | BD bioscience | Cat# 553072, RRID:AB_394604 |
| Anti-CD8-PE | Tonbo Biosciences | Cat# 50-0081, RRID:AB_2621741 |
| Anti-B220-PE | BD bioscience | Cat# 553090, RRID:AB_394620 |
| Anti-NK1.1-PE | BD bioscience | Cat# 553165, RRID:AB_394677 |
| Anti-γδTCR-PE | BD bioscience | Cat# 553178, RRID:AB_394689 |
| Anti-CD11b-PE | BD bioscience | Cat# 557397, RRID:AB_396680 |
| Anti-CD11c-PE | Thermo Fisher Scientific | Cat# 50-0114-80, RRID:AB_11151322 |
| Anti-CD19-PE | Thermo Fisher Scientific | Cat# 50-0193-82, RRID:AB_11218286 |
| Anti-Ter-119-PE | Tonbo Biosciences | Cat# 50-5921, RRID:AB_2621802 |
| EpCAM-FITC | BioLegend | Cat# 118207, RRID:AB_1134106 |
| PDGFRa- PE | Thermo Fisher Scientific | Cat# 12-1401-81, RRID:AB_657615 |
| CD117-APC | Tonbo Biosciences | Cat# 20-1172, RRID:AB_2621587 |
| Anti-CD31-PerCPCy5.5 | BioLegend | Cat#102522,RRID:AB_2566761 |
| Bacterial and Virus Strains | | |
| BL21 Codon plus | Thermo-Fisher Scientific | Cat#C602003 |
|  |  |  |
| Biological Samples |  |  |
| Embryos | C57BL/6 mice | Pregnant mice from Tbx1^+/neo2^ intercrosses |
| Adult and Embryonic Thymus | C57BL/6 mice | Adult and pregnant mice from Tbx1^+/neo2^ intercrosses |
| Tails | C57BL/6 mice | Tbx1^+/neo2^ intercrosses |
| Embryonic Parathyroids, Hearts | C57BL/6 mice | Pregnant mice from Tbx1^+/neo2^ intercrosses |
| Chemicals, Peptides, and Recombinant Proteins | | |
| DAPI | Thermo Fisher Scientific | Cat# D1306, RRID:AB_2629482 |
| Shandon Immu-Mount | Thermo Fisher Scientific | Cat# 9990402 |
| Trypsin | Sigma | Cat# T4049 |
| Critical Commercial Assays | | |
| MesenCult^TM^ Expansion Kit (Mouse) | StemCell Technologies | Cat#05513 |
| Prolong Gold Anti-fade Mountant | Thermo-Fisher Scientific | Cat#P10144 |
| Maxima H Minus cDNA synthesis with dsDNAse | Thermo Scientific | Cat#M1669 |
| Verteporfin | Thermo Scientific | Cat#C816P07 |
| Minoxidil | Sigma | Cat#M4145 |
| Beta- amino propionitrile fumarate | Sigma | Cat#A3134 |
| Labetalol | Sigma | Cat#L1011 |
| Nifedipine | Sigma | Cat#N7635 |
| Enalaprilat | Sigma | Cat#43301 |
| Enalapril maleate | Sigma | Cat#1235300 |
| Prostaglandin E2 | Sigma | Cat#538904 |
| Deposited Data | | |
| GEO -scRNA Seq | 170686 |  |
| GEO -scRNA Seq | In process |  |
| Experimental Models: Organisms/Strains | | |
| Tbx1^+/neo2^ line | Antonio Baldini | CNR Institute of Genetics and Biophysics, Naples |
| C57Bl/J | Jackson Labs | Cat # 000664 |
| Oligonucleotides | | |
| RT PCR Primers, Genotyping Primers |  |  |
| Tbx1 neo2  GCCAGAGGCCACTTGTGTAG | This paper |  |
| Tbx1neo2  GGAGGTCTTCTGGTTTACCCT | This paper |  |
| Tbx1neo2  AGGCTGGGATTCCAAAAGAC | This paper |  |
| Murine Col2a1 F  CAGGATGCCCGAAAATTAGGG | This paper |  |
| Murine Col2a1 R  ACCACGATCACCTCTGGGT | This paper |  |
| Murine Col9a2 F  AAGGGGCCTCCAGGTAAAGTT | This paper |  |
| Murine Col9a2 R  TCCCATTAAACCATCAATGCCA | This paper |  |
| Murine Col11a1 F  CCAGCGGGTCTTATGGGTC | This paper |  |
| Murine Col11a1 R  TGGTAACATCAGCATGGTTCC | This paper |  |
| Murine Sox9 F  CGGAACAGACTCACATCTCTCC | This paper |  |
| Murine Sox9 R  GCTTGCACGTCGGTTTTGG | This paper |  |
| Murine Sox9 F  CGGAACAGACTCACATCTCTCC | This paper |  |
| Murine Acan F  CCTGCTACTTCATCGACCCC | This paper |  |
| Murine Acan R  AGATGCTGTTGACTCGAACCT | This paper |  |
| Murine Pdgfra F  TCCATGCTAGACTCAGAAGTCA | This paper |  |
| Murine Pdgfra R  TCCCGGTGGACACAATTTTTC | This paper |  |
| Murine Gapdh F  AGGTCGGTGTGAACGGATTTG | This paper |  |
| Murine Gapdh R TGTAGACCATGTAGTTGAGGTCA | This paper |  |
|  |  |  |
| Software and Algorithms | | |
| Microsoft Office 365 | Microsoft |  |
| GraphPad Prism | GraphPad Software, Inc. | Prism 9.1 |
| SnapGene | GSL Biotech LLC | SnapGene 5.2.4 |
| PyMol molecular visualization software | Schrodinger, Inc. | PyMol 2.4 |
| Keyence BT software | Keyence Corp. | BT W |
| FlowJo flow cytometry software | TreeStar Inc. | Flowjo 10.6.1 |
| Image J | NIH source software | https://imagej.nih.gov/ij/ |
| Cell ranger | 10X Genomics | Cell Ranger 3.0.0 |
